# Colonization and Succession within the Human Gut Microbiome by Archaea, Bacteria, and Microeukaryotes during the First Year of Life

**DOI:** 10.3389/fmicb.2017.00738

**Published:** 2017-05-02

**Authors:** Linda Wampach, Anna Heintz-Buschart, Angela Hogan, Emilie E. L. Muller, Shaman Narayanasamy, Cedric C. Laczny, Luisa W. Hugerth, Lutz Bindl, Jean Bottu, Anders F. Andersson, Carine de Beaufort, Paul Wilmes

**Affiliations:** ^1^Luxembourg Centre for Systems Biomedicine, University of LuxembourgEsch-sur-Alzette, Luxembourg; ^2^Integrated BioBank of LuxembourgLuxembourg, Luxembourg; ^3^Science for Life Laboratory, Division of Gene Technology, School of Biotechnology, Royal Institute of TechnologyStockholm, Sweden; ^4^Centre Hospitalier de LuxembourgLuxembourg, Luxembourg

**Keywords:** fungi, succession, delivery mode, infant gut microbiome, amplicon sequencing, microbial colonization, quantitative real-time PCR

## Abstract

Perturbations to the colonization process of the human gastrointestinal tract have been suggested to result in adverse health effects later in life. Although much research has been performed on bacterial colonization and succession, much less is known about the other two domains of life, archaea, and eukaryotes. Here we describe colonization and succession by bacteria, archaea and microeukaryotes during the first year of life (samples collected around days 1, 3, 5, 28, 150, and 365) within the gastrointestinal tract of infants delivered either vaginally or by cesarean section and using a combination of quantitative real-time PCR as well as 16S and 18S rRNA gene amplicon sequencing. Sequences from organisms belonging to all three domains of life were detectable in all of the collected meconium samples. The microeukaryotic community composition fluctuated strongly over time and early diversification was delayed in infants receiving formula milk. Cesarean section-delivered (CSD) infants experienced a delay in colonization and succession, which was observed for all three domains of life. Shifts in prokaryotic succession in CSD infants compared to vaginally delivered (VD) infants were apparent as early as days 3 and 5, which were characterized by increased relative abundances of the genera *Streptococcus* and *Staphylococcus*, and a decrease in relative abundance for the genera *Bifidobacterium* and *Bacteroides*. Generally, a depletion in Bacteroidetes was detected as early as day 5 *postpartum* in CSD infants, causing a significantly increased Firmicutes/Bacteroidetes ratio between days 5 and 150 when compared to VD infants. Although the delivery mode appeared to have the strongest influence on differences between the infants, other factors such as a younger gestational age or maternal antibiotics intake likely contributed to the observed patterns as well. Our findings complement previous observations of a delay in colonization and succession of CSD infants, which affects not only bacteria but also archaea and microeukaryotes. This further highlights the need for resolving bacterial, archaeal, and microeukaryotic dynamics in future longitudinal studies of microbial colonization and succession within the neonatal gastrointestinal tract.

## Introduction

The human microbiome contributes essential functionalities to human physiology and is thought to play a crucial role in governing human health and disease (Greenhalgh et al., 2016). A growing body of evidence suggests that chronic diseases such as allergies (Abrahamsson et al., 2012, 2014), type 2 diabetes (Delzenne et al., 2015), obesity (Turnbaugh et al., 2006), and metabolic syndrome (Vrieze et al., 2012) are associated with a disequilibrium in the microbiome of the human gastrointestinal tract (GIT).

The initial microbiome colonization process is crucial for the development and maturation of the GIT as well as the immune system of the developing infant (Björkstén, 2004; Caicedo et al., 2005; Rautava and Walker, 2007; Eberl and Lochner, 2009; Houghteling and Walker, 2015). During vaginal delivery, a subset of the maternal bacterial community is supposedly transferred to the infant; in contrast, early-stage microbiome profiles from infants delivered by cesarean section (C-section) are typically not as reflective of the mothers' vaginal or gastrointestinal environment (Dominguez-Bello et al., 2010; Bäckhed et al., 2015; Nayfach et al., 2016). Based on spatio-temporal studies in humans (Abrahamsson et al., 2014), it has been suggested that various disturbances in the initial microbiome colonization process as early as 1 month after birth may increase chronic disease susceptibilities over the course of human life (Arrieta et al., 2014; Cox et al., 2014; Houghteling and Walker, 2015). It has been previously observed that the delivery mode is the most important factor in determining the early colonization pattern(s) (Biasucci et al., 2008; Dominguez-Bello et al., 2010; Jakobsson et al., 2014; Rutayisire et al., 2016), although other factors, such as diet (breast milk vs. formula milk; Le Huërou-Luron et al., 2010), gestational age (term delivery vs. preterm delivery; Barrett et al., 2013), or the maternal intake of antibiotics (Sekirov et al., 2008) have also been observed to have effects on this process.

Even though the colonization and succession within the GIT have been studied extensively, the focus has mostly been directed to the bacterial domain. However, such a constrained view may lead to an underestimation of the contribution of the archaeal and eukaryotic domains, in particular microeukaryotes, such as unicellular parasites or yeasts, and could ultimately lead to incomplete conclusions (Horz, 2015).

Within the archaeal domain, methanogenic archaea (mainly those belonging to the order Methanobacteriales) have been estimated to comprise between 10^8^ and 10^10^ cells per gram dry weight of stool (Miller and Wolin, 1986) and are considered almost ubiquitous inhabitants of the intestinal microbiome with a presence in up to 95.7% of all adult humans (Dridi et al., 2009). Methanogenic archaea are functionally important due to their ability to consume molecular hydrogen, which is both an end product and a concentration-dependent inhibitor of bacterial fermentation (Thauer et al., 2008). Consequently, methanogens drive the effective degradation of organic substances and play an important role in interspecies hydrogen transfer through maintaining syntrophic relationships with bacterial populations (Hansen et al., 2011). Additionally, gut methanogens have been linked to energy metabolism and adipose tissue deposition of the human host (Samuel et al., 2007), and the ability of certain archaea to produce methane may play a role in the pathogenesis of several intestinal disorders (Roccarina et al., 2010). Despite these observations, the simultaneous presence of archaea and bacteria has been ignored in the majority of studies on the gastrointestinal microbiome to date and details about neonatal colonization by archaea remain limited. Previous studies have detected archaea transiently and almost exclusively in the first few weeks of life, and considerably less in samples collected after the fifth week of life (Palmer et al., 2007). Archaea have been sporadically detected in the vaginal environment before, although exclusively in women with bacterial vaginosis (Belay et al., 1990). As archaea are mainly inhabitants of the human GIT, but also colonize the skin surface (Probst et al., 2013) as well as the oral cavity (Nguyen-Hieu et al., 2013), a transfer from mother to infant by fecal-oral or oral-oral route seems thereby most probable.

Eukaryotes and microeukaryotes, which form part of the human microbiota, have been shown to exert immunomodulatory effects on the host (Weinstock, 2012; Rizzetto et al., 2014). Furthermore, infections by parasitic eukaryotes have been linked to decreased allergic and autoimmune disease prevalence (Weinstock, 2012) and have been used for therapeutic interventions in that context (McFarland and Bernasconi, 1993; Williamson et al., 2016). However, the role of microeukaryotes within the human GIT microbiome and the resulting impact on the human host remain so far unresolved (Andersen et al., 2013). It has been previously reported that the overall microeukaryotic diversity of the adult human GIT is low but largely temporally stable (Scanlan and Marchesi, 2008), whereas other research suggested that the adult GIT microbiome harbors a complex microeukaryotic community with the most abundant taxa by far being fungi (Hamad et al., 2012). To date, a single study followed the initial colonization of the GIT by microeukaryotes using 18S rRNA gene amplicon sequencing in four newborn infants (Pandey et al., 2012), but failed to detect any microeukaryotes at the timepoints analyzed. However, this study might have been substantially limited by its sample collection as well as the applied sequencing technique.

In our present work, a longitudinal study was conducted to describe the colonization and succession of the three domains of life within the GIT of newborns. More specifically, we investigated the microbiome changes during the first year of life among eight vaginally delivered (VD) infants and seven infants delivered by C-section (CSD). The latter are statistically at a higher risk of developing metabolic disease such as obesity (Mueller et al., 2015) and/or related diseases like type 2 diabetes (Nguyen and El-Serag, 2010), as well as allergic diseases such as atopic eczema (Abrahamsson et al., 2012) and asthma (Abrahamsson et al., 2014) in childhood and/or adulthood. Fecal samples were collected from all infants (VD and CSD) at six time points between day 1 and 1 year *postpartum* and, using quantitative real-time PCR (qPCR), we determined the sizes of prokaryotic (bacteria and archaea) and fungal populations, the relative quantities of archaea and validated the amounts of four selected bacterial genera and two phyla in the collected samples. Additionally, targeted high-throughput 16S and 18S rRNA gene amplicon sequencing was conducted on the isolated DNA. After processing and filtering of the resulting data, we compared the prokaryotic and microeukaryotic community structures in relation to the delivery mode and a multitude of other recorded maternal/neonatal characteristics. The resulting data provides a detailed overview of the neonatal colonization and succession patterns of members of all three domains of life.

## Materials and methods

### Sample collection, processing, and biomolecular extraction

#### Study context

In the context of the national COSMIC study, pregnant women were recruited in Luxembourg starting in 2012. The 15 pregnant women included in the presented study were aged between 24 and 42 years and gave birth in the maternity department of the Centre Hospitalier de Luxembourg (CHL). This study was carried out in accordance with the recommendations of good clinical practices established by the “International Council for Harmonization of Technical Requirements for Pharmaceuticals for Human Use” with written informed consent from all subjects in accordance with the Declaration of Helsinki. The protocol and informed consent form was approved by the Luxembourg “Comité National d'Ethique de Recherche” in 2011 (reference number 201110/06).

#### Sample and data collection

To mitigate pre-analytical confounders, fecal samples were immediately snap-frozen in liquid nitrogen or placed on dry ice following collection and were stored at −80°C until further processing. Fecal samples were scheduled to be collected at day 1, 3, 5, 28, 150, and 365. The medical histories of both parents and medication intake of the mother were recorded, as well as weight, date of birth, gender, mode of delivery, and gestational age of the infant. Additional data, which was collected subsequently for all infants included weight, type of milk fed, medication intake including antibiotics and time point at which solid food was introduced. If an infant received formula at a specific point in time, it was considered as receiving combined feeding for the entire remainder of the study, as even short-term formula-feeding has been shown to cause profound and long lasting shifts to the gastrointestinal microbiome composition (Guaraldi and Salvatori, 2012). Hospitalization in the neonatal care unit and administration of antibiotics to infants immediately *postpartum* as well as birth prior to 34 weeks of gestation were exclusion criteria. Samples and associated data were collected and stored at the Integrated BioBank of Luxembourg (IBBL) following ISO17025:2005 standards and the International Society for Biological and Environmental Repositories (ISBER) best practices.

#### DNA extraction from fecal samples

Pre-processing of all fecal samples (150–200 mg of weighed material) was carried out according to Shah et al. (in press; subsection 3.2, steps 1–4). After high-speed centrifugation, DNA was extracted from the resulting interphase pellet using the PowerSoil® DNA isolation kit (MOBIO Laboratories, Belgium). The method was optimized for mechanical disruption with bead-beating to ensure a realistic representation of microbial communities (Walker et al., 2015). DNA quality and quantity were determined on 1% agarose gels, by NanoDrop 2000c spectrophotometer (Thermo Fisher Scientific, USA) and Qubit 2.0 fluorometer (Thermo Fisher Scientific, USA). The extracted DNA was stored at −80°C until qPCR validation and sequencing library construction.

### DNA analyses and sequencing

#### Quantitative real-time PCR

Extracted DNA was diluted, when applicable, to a concentration of 5 ng/μl and amplified in duplicates, using previously published primers targeting prokaryotes, archaea, or specific fungi as well as specific bacterial genera and phyla (Table 1), which were ordered and received from Eurogentec (Belgium). The reaction mixture contained 1 μl template DNA, 5 μl of Mastermix (iQ SYBR Green Supermix; Bio-Rad Laboratories, USA), and 500 nMol of each primer, in a final reaction volume of 10 μl. Genomic DNA isolated from *Salmonella* Typhimurium LT2 and *Saccharomyces cerevisiae* BY4743 was used to prepare standard curves for the universal prokaryotic and fungal primers, respectively. A sample pool, comprised of 1 μl of undiluted DNA from each of the 65 samples, was used to prepare standard curves for all assays. All standard curves were prepared with a total of at least five successive 10-fold dilutions. qPCR was performed on a LightCycler 480 (Roche Diagnostics, Germany) with an initial denaturation step of 1 min at 95°C followed by primer-specific cycling times (Table 1), a single fluorescence acquisition step at the end of each extension step and a final melting curve. Crossing point (Cp) values were calculated using the second derivative method within the Roche LightCycler 480 software version 1.5. Absolute copy numbers of prokaryotic 16S and fungal 18S rRNA genes were calculated using the Cp values and the reaction efficiencies based on the standard curves obtained from defined DNA samples and extractions yields were estimated from these numbers. Relative concentrations of specific taxa compared to all 16S rRNA genes were calculated using Cp values and the standard curves obtained for the sample pool. Only samples where the target was positively detected in both duplicate reactions were considered for further analyses.

**Table 1:** **Primer pairs and conditions of quantitative real-time PCR**.

**Main target (target gene)**	**Designation**	**Oligonucleotide sequence (5′-> 3′)**	**Annealing temperature (°C)**	**Cycling**	**References**
Fungi (18S rRNA)	Fungi2F Fungi2R	F: ATT-GGA-GGG-CAA-GTC-TGG-TG R: CCG-ATC-CCT-AGT-CGG-CAT-AG	55	60 cycles: 15 s at 95°C, 10 s at 55°C, 25 s at 72°C	Einsele et al., 1997
*Staphylococcus* (tuf)	TStaG422-F TStag765-R	F: GGC-CGT-GTT-GAA-CGT-GGT-CAA-ATC-A R: TAT-HAC-CAT-TTC-AGT-ACC-TTC-TGG-TAA	55	45 cycles: 20 s at 95°C, 30 s at 55°C, 1 min at 72°C	Martineau et al., 2001
*Haemophilus* (P6)	HI-IV	F: ACT-TTT-GGC-GGT-TAC-TCT-GT	55		van Ketel et al., 1990
	HI-V	R: TGT-GCC-TAA-TTT-ACC-AGC-AT			
Universal archaea (16S rRNA)	ARC787F ARC1059R	F: ATT-AGA-TAC-CCS-BGT-AGT-CC R: GCC-ATG-CAC-CWC-CTC-T	60		Yu et al., 2005
*Lactobacillus* (16S rRNA)	Lac774F Lac989R	F: GCG-GTG-AAA-TTC-CAA-ACGR: GGG-ACC-TTA-ACT-GGT-GAT	60	45 cycles: 15 s at 95°C, 30 s at 60°C, 1 min at 72°C	Hermann-Bank et al., 2013
*Streptococcus* (16S rRNA)	Strep488F	F: CTW-ACC-AGA-AAG-GGA-CGG-CT	60		Hermann-Bank et al., 2013
	Strep824R	R: AAG-GRY-CYA-ACA-CCT-AGC			
Firmicutes (16S rRNA)	Lgc353	F: GCA-GTA-GGG-AAT-CTT-CCG	60		Fierer et al., 2005
	Eub518	R: ATT-ACC-GCG-GCT-GCT-GG			
Bacteroidetes (16S rRNA)	798cfbF cfb967R	F: CRA-ACA-GGA-TTA-GAT-ACC-CT R: GGT-AAG-GTT-CCT-CGC-GTA-T	61	45 cycles: 15 s at 95°C, 20 s at 61°C, 30 s at 72°C	Bacchetti De Gregoris et al., 2011
Universal prokaryotes (16S rRNA)	926F	F: AAA-CTC-AAA-KGA-ATT-GAC-GG	61		Bacchetti De Gregoris et al., 2011
	1062R	R: CTC-ACR-RCA-CGA-GCT-GAC			

#### 16S/18S rRNA gene amplicon sequencing

Specific sets of primers targeting 16S and 18S rRNA genes were chosen for the amplification and subsequent sequencing to broadly cover bacterial, archaeal and eukaryotic diversity. The bacterial and archaeal community structures of the 65 samples were resolved by amplifying the V4 region of the 16S rRNA gene using the universal primers 515F and 805R (515F_GTGBCAGCMGCCGCGGTAA; 805R_GACTACHVGGGTATCTAATCC; Herlemann et al., 2011; Hugerth et al., 2014a). This primer pair covers the bacterial domain, including the phylum Actinobacteria and additionally resolves the archaeal domain.

The eukaryotic community structures for 63 samples were analyzed by amplifying the V4 region of the 18S rRNA gene using primers 574^*^F and 1132R (574^*^F_CGGTAAYTCCAGCTCYV; 1084r_CCGTCAATTHCTTYAART; Hugerth et al., 2014b). Two samples did not yield sufficient amplicons (CSD infant 7 collected on days 1 and 3).

The KAPA HiFi HotStart ReadyMix (Kapa Biosystems, Wilmington, MA, USA) was used for amplification with 25 cycles and according to the service provider's standards. Paired-end sequencing with 2 × 300 nt was performed on an Illumina MiSeq platform with the V3 MiSeq kit at the Center of Analytical Research and Technology—Groupe Interdisciplinaire de Génoprotéomique Appliquée (CART-GIGA; Liège, Belgium).

#### 16S rRNA and 18S rRNA gene sequencing data processing

The raw 16S rRNA gene amplicon sequencing data were processed using the LotuS software (version 1.35) with default parameters (Hildebrand et al., 2014). After clustering the reads into operational taxonomic units (OTUs) at 97% identity level, they were classified and taxonomically assigned using the Ribosomal Database Project (RDP) classifier version 2.10.1 (Wang et al., 2007). OTUs with a confidence level below 0.8 at the domain level were discarded. The amplicon sequences belonging to the 100 most abundant OTUs were additionally manually curated for unspecific amplification. As only few archaeal reads were detected, the overall quality of the archaeal reads were manually assessed using the FASTQC results^1^. As the paired-end 18S rRNA gene amplicon reads obtained in this study did not overlap, a specifically tailored workflow was used to process the raw 18S rRNA gene amplicon sequencing data^2^. For the classification step and the taxonomic assignment, the PR2 database (Guillou et al., 2013) was used according to Hu et al. (2016).

#### 16S rRNA and 18S rRNA gene sequencing data analysis

In order to exclude sequencing artifacts for both prokaryotic and eukaryotic datasets, we removed OTUs that were represented by <10 reads in all of the sequenced samples, thereby examining the dominant phylotypes for all three domains of life throughout this study. One sample was excluded from further analyses as its read count (4,141 reads) was far below the average read count of 213,469.5 ± 84,713.4 reads (average ± standard deviation) for all 16S rRNA gene sequencing datasets and was thereby yielding <5,000 16S rRNA gene amplicon reads that are necessary for assessing bacterial diversity (Lundin et al., 2012; Kozich et al., 2013; Song et al., 2013; Sebald et al., 2016; Hill-Burns et al., 2017). As the complexity of the microeukaryotic community structure is largely undetermined and no previous recommendations exist, no cutoff for the number of 18S rRNA gene amplicon reads was applied. All statistical analyses and visualizations were performed using the R statistical software package (version 3.2.0; R Development Core Team, 2008). Per-sample normalization, calculations of richness, diversity (Shannon's diversity index), evenness (Pielou's evenness index), dissimilarity index (distance to the most mature sample, calculated using Soerensen's similarity index of presence/absence of taxa at each individual time point compared to samples collected at the last individual time point) and non-parametric estimation of minimum community richness according to Chao (1984) were performed using the “vegan” package^3^. For the calculations of diversity and evenness indices for microeukaryotes, only samples with a total of more than 10 reads were considered. Differential analysis of relative OTU abundances based on read count data for the 16S rRNA gene amplicon sequencing dataset was done using the “DESeq2” package (Love et al., 2014), which allows testing for differential abundance using negative binomial generalized linear models and multiple-testing adjustment by controlling the false discovery rate (Benjamini and Hochberg, 1995). Adobe Illustrator (version 19.1.0) was used for labeling axes and creating multi-plot graphs.

Various neonatal characteristics that were previously shown to have an impact on the microbiome (e.g., delivery mode, fed milk type, gestational age, maternal antibiotic, and probiotic intake, positive screening for Group B *Streptococcus* (*Streptococcus agalactiae*) colonization of the mother) were compared between samples using the Wilcoxon rank sum test or Kruskal–Wallis test where applicable and comparisons with *P* < 0.05 were considered statistically significant. Principal coordinate analysis (PCoA) graphs were generated using the Jensen-Shannon distance as implemented in the R package “phyloseq” (McMurdie and Holmes, 2013) and clusters were defined using the partitioning around medoids (pam) function contained in the R package “cluster” (Maechler et al., 2016).

#### Workflow for DNA mock extracts and control samples

As negative controls for the qPCR quantifications and 16S/18S rRNA amplicon sequencing, sample-free “DNA mock extracts,” i.e., 2 extraction controls that underwent the same DNA extraction protocol without initial input, and a no template control, were prepared and subjected to qPCR analyses and amplicon sequencing together with the study samples.

In order to exclude any biases by low-yield samples (Salter et al., 2014; Jervis-Bardy et al., 2015), a control fecal sample from a single healthy female adult individual was collected and preserved under the same conditions as described. This control stool sample was extracted using the same DNA extraction protocol and created a dilution series ranging from 2 to 0.002 ng/μl. The four DNA dilution samples were 16S rRNA gene amplicon sequenced using the same primer pair as for the collected study samples.

## Results

### Cohort characteristics

Sixty-five fecal samples were collected between September 2012 and April 2014 at the CHL from eight healthy VD and seven healthy CSD infants at six time points (samples collected around days 1, 3, 5, 28, 150, and 365). The birth weights as well as the gestational ages of the infants were similar, while the ratios of genders, the maternal age and the maternal postnatal body mass index (BMI) differed between both groups, with the CSD group comprising more male infants as well as mothers with a higher average age and postnatal BMI (Table 2). Three mothers who gave birth vaginally screened positively for Group B *Streptococcus*, whereas all mothers giving birth by C-section were screened negatively. Clinical healthcare guidelines in Luxembourg recommend that mothers who were screened positively for Group B *Streptococcus* should be treated intravenously with antibiotics prior to birth. Although mothers undergoing C-section were preferentially treated with antibiotics prior to birth, the majorities of both cohorts received antibiotic treatment (Table 2). Two of the three mothers who did not receive any antibiotics prior to birth chose to take probiotics during their pregnancies, whereas none of the other mothers recorded any probiotic supplementation. Out of eight VD infants, four were fed purely with maternal breast milk, while two others received formula milk and the remaining two were fed a combination of formula and breast milk. Out of the seven CSD infants, five were purely fed breast milk and the remaining two received a combination of breast milk and formula (Supplementary File 1, Table S1). According to the self-assessment of mothers that were purely breastfeeding, both the frequency and duration of feeding were not significantly different between VD and CSD infants. Introduction of solid food occurred in average around day 150 for all infants.

**Table 2:** **Neonatal and maternal characteristics (***n*** = 15)**.

	**Total cohort (*n* = 15)**	**VD (*n* = 8)**	**CSD (*n* = 7)^a^**
**INFANT CHARACTERISTICS**			
Female gender	7 (46.7%)	5 (62.5%)	2 (28.6%)
Gestational age at delivery (weeks)	38.7 ± 1.8	39 ± 1.5	38.3 ± 2.1
Birth weight (g)	3273 ± 416	3311 ± 543	3230 ± 236
**MATERNAL CHARACTERISTICS**			
Positive group B *Streptococcus* screening	3 (21.4%)	3 (37.5%)	0
Age	33.6 ± 4.6	32.5 ± 4.4	35 ± 4.8
Postnatal body mass index	24 ± 4.3	21.8 ± 2.7	26.8 ± 4.6
**ETHNICITY**			
Caucasian	12 (85.7%)	7 (87.5%)	5 (83.3%)
African	2 (14.3%)	1 (12.5%)	1 (16.7%)
Perinatal antibiotic intake^b^	11 (78.6%)	6 (75%)	5 (83.3%)
Penicillin^c^	6 (42.9%)	6 (75%)	0
Cephalosporin	4 (28.6%)	0	4 (66.7%)
Clindamycin	1 (7.1%)	0	1 (16.7%)
Probiotic use during pregnancy	2 (14.3%)	1 (12.5%)	1 (16.7%)

a *2 C-section infants are twins*.

b *Considering all antibiotics administered to the mother 12 h prior and after the delivery*.

c As ampicillin belongs to the penicillin group, ampicillin and penicillin intake were both categorized as “penicillin.”

*Study groups are defined according to delivery mode (VD: n = 8; CSD: n = 7). CSD, C-section delivery; VD, vaginal delivery*.

### Assessment of bacterial, fungal, and archaeal load using real-time PCR

**Figure 1:**
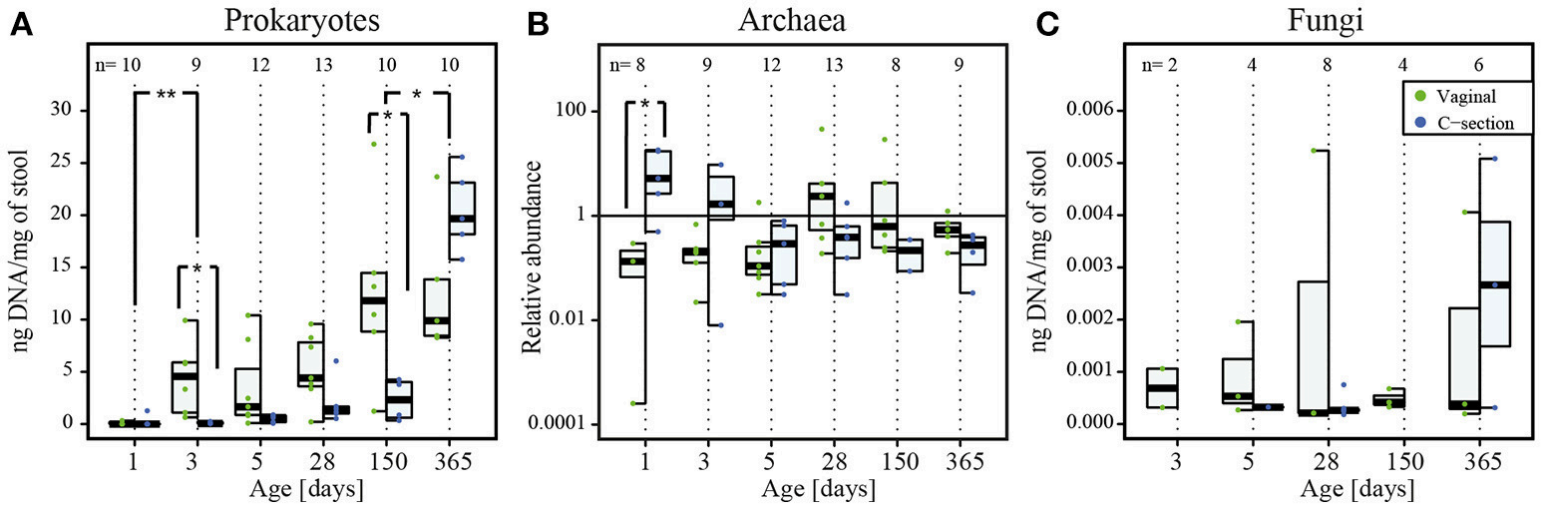
**Detection of prokaryotes, archaea and fungi in infant stool during the first year of life. (A)** Absolute quantification of 16S rRNA gene copy numbers for prokaryotic DNA (ng DNA per mg of stool), **(B)** relative quantification of archaeal read counts. **(C)** Absolute quantification of 18S rRNA gene copy numbers for fungal DNA (ng DNA per mg of stool) by quantitative real-time PCR and over the course of the first year of life. The numbers of samples per collection time point are provided at the top of the graph. For the purpose of clarity, only significant differences between subsequent time points are shown in the figure; for all significant differences between collection time points, see Supplementary File 1, Tables S3, S4. Significant differences obtained by Wilcoxon rank sum test between consecutive time points are represented by asterisks (^*^ when *P* < 0.05; ^**^ when *P* < 0.01). CSD, C-section delivery; VD, vaginal delivery. Fecal samples originating from VD infants are represented on the left side of each barplot and by green points, samples from CSD infants are represented on the right side of each barplot and by blue points.

Specific qPCR assays using previously published primers were used to obtain quantitative information on the individual taxonomic groups of interest (Table 1). Absolute yields of extracted DNA were quantified and prokaryotic and fungal DNA, as well as the relative quantities of archaea were calculated based on the ratio between the relative concentrations obtained for the universal prokaryotic primer pair and the relative concentrations obtained for archaea (Figure 1). The detection of organisms in the “DNA mock extracts” reflecting the three domains of life was negative for the archaea- and fungi-specific primer sets whereas the universal prokaryotic primer set resulted in the detection of a minimal amount of DNA close to the qPCR detection limit (average concentration of 0.002 ng/μl measured for the “DNA mock extracts” as opposed to 0.3 ng/μl measured for meconium samples, i.e., the earliest fecal material excreted by infants, which had the lowest observed concentrations amongst all study samples). Therefore, the mock extracts and subsequent analyses did not indicate the presence of reagent-derived contaminants.

The qPCR-based quantification of prokaryotic DNA was successful for 64 out of 65 samples, with yields ranging from 0.2 ± 0.4 ng of DNA per mg of stool (average ± standard deviation) in the meconium samples of day 1, to 16.6 ± 6.4 ng of DNA per mg of stool on day 365. Generally, the prokaryotic load of both cohorts increased considerably after the introduction of food. The DNA yields were dependent on the collection time point, and the greatest differences were observed between day 1 and all other collection time points (Figure 1A; for all significant differences between collection time points, see Supplementary File 1, Table S2). Moreover, at day 5 significantly lower extraction yields (*P* = 0.03; Wilcoxon rank sum test) were observed for samples derived from infants whose mothers received antibiotics prior to birth (Supplementary File 1, Figure S1).

The presence of archaea was detected in 91% of all samples (59 out of 65 samples) and the concentration of archaeal DNA relative to the mean of all samples ranged from 5.5 ± 7.8 on day 1 to 0.5 ± 0.4 on day 365. Generally, more samples were found to be positive in VD (97% of VD infant samples) than in CSD infants (86% of CSD infant samples) and archaeal presence was as well-detected in the samples from the very first time points (Figure 1B).

Presence of fungal organisms was detected in 37% (24 out of 65 samples) of all samples, ranging from 0.0007 ± 0.0005 ng of fungal DNA per mg of stool on day 3 to 0.002 ± 0.002 ng of fungal DNA per mg of stool on day 365, with generally more samples being positive for fungi in VD (43% of VD infant samples) compared to CSD infants (31% CSD infant samples). Fungi were detected earliest at day 3 in VD and at day 5 in CSD infants. The fungal DNA yield tended to increase over time, even though the magnitude of the increase was smaller compared to prokaryotes (Figure 1C).

### Validation of GIT microbiome profiles in low-yield samples

The absolute quantification of prokaryotic 16S rRNA gene copy numbers in all samples showed that the earliest samples contained significantly less microbial DNA compared to all other visits (Figure 1, Supplementary File 1, Table S2).

Analyzing the 16S rRNA gene sequencing data obtained for the dilution series of the human adult fecal control sample, we observed that the undiluted sample, reflecting the concentration of most samples in the study (Figure 1), and all three dilutions, simulating low-yield samples, showed highly comparable diversity and evenness indices (Supplementary File 1, Figure S2A). For richness, the undiluted sample and both 10- and 100-fold diluted samples had highly comparable results, while the 1,000-fold dilution caused a slight decrease. This loss of observed richness is also reflected in a slightly increased dissimilarity index for the 1,000-fold diluted sample compared to the undiluted sample. Considering the observed taxonomic composition with decreasing DNA concentration, all three dilutions showed high resemblance to the undiluted sample, while the 100- and 1,000-fold dilutions showed slightly over-estimated relative abundances for *Roseburia* spp. and *Collinsella* spp. and a slight under-estimation for *Bacteroides* spp. (Supplementary File 1, Figure S2B). However, in each case, a similar taxonomic profile to the one in the undiluted sample was observed and potential reagent contaminants or sequencing artifacts did not have a significant effect on the taxonomic composition in the low-yield samples. These data indicated that the chosen approach allowed the comparison of samples with low extraction yields to those with higher yields.

### Generated amplicon sequencing data

After the 16S rRNA gene sequencing and following the primary data processing and filtering, a total of 13,136,451 reads were retained and used for the subsequent analyses. With 205,000 ± 90,000 reads per sample (average ± standard deviation), a total of 1,053 unique OTUs were identified. One out of the 65 samples was excluded from further 16S rRNA gene sequencing analysis due to poor coverage (sample collected at day 3 for VD infant 8).

For the processed 18S rRNA gene amplicon sequence data, only OTUs reflecting the microeukaryotic members of the microbiome were considered. To achieve this, we manually curated the dataset of initially 3,376,004 reads by removing classified OTUs that belonged to the following clades containing multicellular organisms: Metazoa (total of 3,302,231 reads), Chlorophyta (total of 4,611 reads), Streptophyta (total of 7,414 reads), and Agaricomycetes (7,038 reads). After filtering out OTUs that were represented by <10 reads, a total of 60,476 reads (average of 960 ± 1,540 reads per sample) and 152 microeukaryotic OTUs were retained for the subsequent analyses.

### Prominent bacterial, archaeal, and microeukaryotic taxa

**Figure 2:**
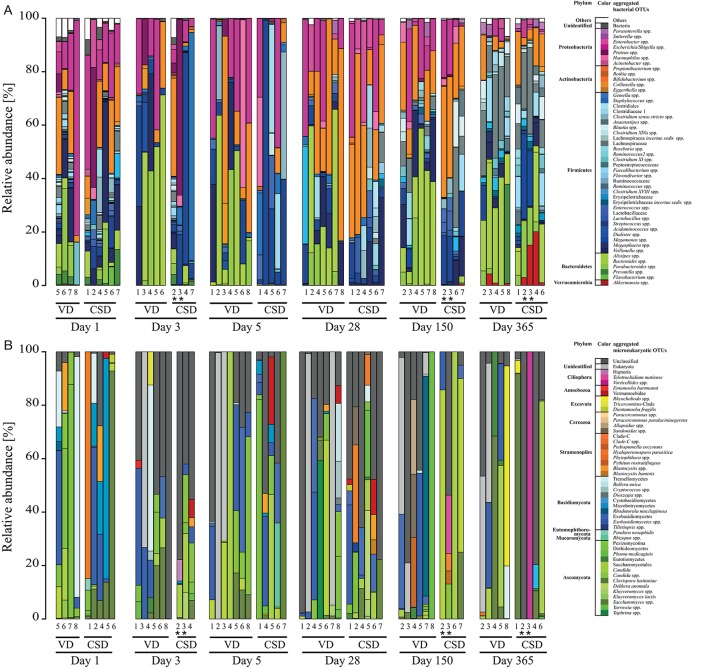
**Prokaryotic and microeukaryotic microbiome compositions in infants over the first year of life**. Barplots of relative abundances of the 49 most abundant taxa per sample for **(A)** prokaryotes and **(B)** microeukaryotes for both delivery modes. All OTUs with the same taxonomy were regroupedd into the same taxa, whereas taxa that did not belong to the 49 most abundant were regrouped under “Others.” Sequences were classified to the highest taxonomic level that could be confidently assigned. Aggregated OTUs are color-coded according to the phylum they belong to. Numbers below the barplots are representative of the different infants in the study. CSD, C-section delivery; VD, vaginal delivery. ^*^Twins.

In order to resolve which specific taxa were present during neonatal GIT colonization, we first identified the most common and abundant OTUs in the 16S rRNA gene amplicon sequencing data, which belonged to the phyla Proteobacteria, Actinobacteria, Firmicutes, Bacteroidetes, and Verrucomicrobia (Figure 2A). Bacterial genera present in all samples (“core populations”) included *Bifidobacterium* spp., *Escherichia/Shigella* spp., *Bacteroides* spp., *Streptococcus* spp., and *Enterococcus* spp., with the first three genera also being the bacterial taxa represented by the most reads out of the total of sequencing reads in all samples (Supplementary File 2).

As our qPCR results suggested the presence of archaea in most samples, their classification was scrutinized by 16S rRNA gene sequencing. Two OTUs belonging to the domain archaea were identified. OTU 1128 was assigned to the genus *Methanosphaera* and comprised a total of 25 reads in a single sample (day 1 for a VD5, 0.02% of reads; Supplementary File 2). Despite being low in abundance, reads of OTU 1128 (*Methanosphaera* sp.) were of good quality and allowed us to confidently ascertain the presence of this organism in this sample (Supplementary File 1, Figures S3A,B). Meanwhile, OTU 693, assigned to the genus *Methanobrevibacter*, was found in four samples represented by 1–11 reads but showed insufficient sequence quality for a confident classification (Supplementary File 1, Figures S3C,D).

Overall, microeukaryotic taxa were less frequent in the individual samples compared to bacterial taxa, with fewer OTUs and without specific “core” OTUs, which were detected in all samples. The most represented fungal phyla in all samples belonged to the phyla Basidiomycota and Ascomycota (Figure 2B), with the genus *Saccharomyces* and the class Exobasidiomycetes having been detected in more than 40% of the samples (Supplementary File 3).

Interestingly, meconium samples already presented a relatively large diversity of different prokaryotic and microeukaryotic populations. For prokaryotes, a total of 674 OTUs were detected in the 10 collected meconium samples (minimum of 109 OTUs, maximum of 347; Supplementary File 4). OTUs that were detected in all meconium samples included *Escherichia/Shigella* spp. and *Bifidobacterium* spp., which were also two of the taxa with the highest read counts over all samples. *Enterobacter* spp., *Staphylococcus* spp., *Streptococcus* spp., *Veillonella* spp., *Bacteroides* spp., *Prevotella* spp., *Clostridium sensu stricto* spp., *Delftia* spp., and *Blautia* spp. were also detected across all meconium samples. For the microeukaryotic community, a total of 45 OTUs were detected in the 9 sequenced meconium samples (Supplementary File 5). The most frequently detected OTU (in 77.8% of meconium samples) belonged to *Exobasidiomycetes* spp., while *Saccharomyces* spp., represented by the two most dominant OTUs with the highest relative abundances, were detected in more than half of the meconium samples.

### Colonization and succession

**Figure 3:**
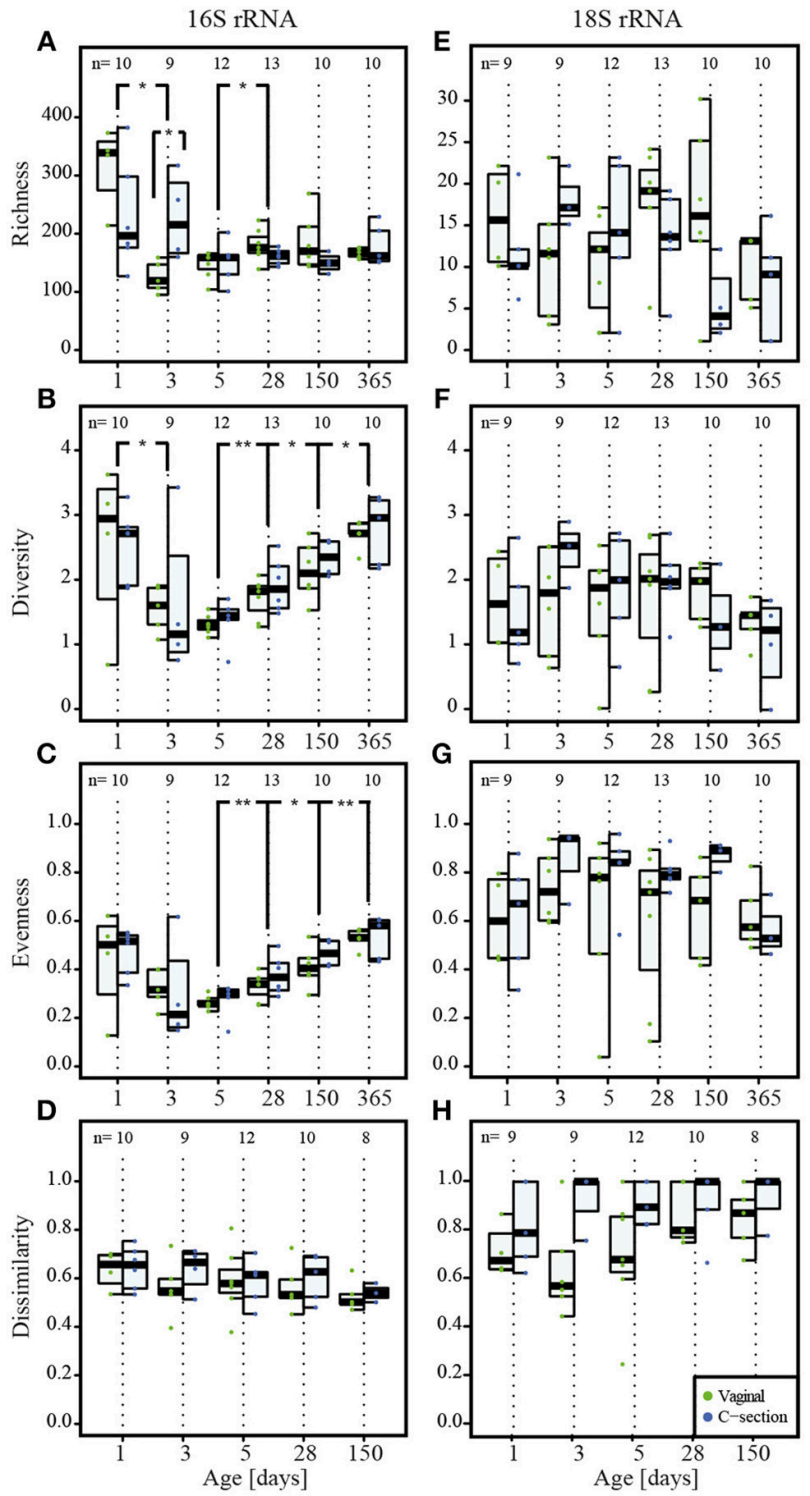
**Colonization of prokaryotes and microeukaryotes**. Depiction of **(A,E)** richness (number of OTUs), **(B,F)** diversity (Shannon's diversity index), **(C,G)** evenness (Pielou's evenness index) and **(D,H)** dissimilarity index reflecting the distance to the most mature sample (Soerensen's similarity index of presence/absence of taxa at each individual time point compared to the most mature microbial community structures represented by samples collected at the last individual time point) for prokaryotes and microeukaryotes, respectively. Dominant phylotypes for prokaryotic and microeukaryotic datasets were considered. The numbers of samples per collection time point are provided at the top of the graph. For the purpose of clarity, only significant differences as assessed by Wilcoxon rank sum test between subsequent time points are shown in the figure; for all significant differences between collection time points, see Supplementary File 1, Table S3 for the prokaryotic and Table S4 for the microeukaryotic datasets. Significant differences between consecutive time points are represented by asterisks (^*^ when *P* < 0.05; ^**^ when *P* < 0.01). CSD, C-section delivery; VD, vaginal delivery. Fecal samples originating from VD infants are represented on the left side of each barplot and by green points, samples from CSD infants are represented on the right side of each barplot and by blue points.

As the amount of microbial DNA in the infants' stool increased with time, we analyzed whether the increase in microbial biomass was accompanied by a change in community characteristics such as richness or diversity. Based on the 16S and 18S rRNA gene amplicon data, we calculated overall richness, diversity, evenness, and dissimilarity indices for the prokaryotic (bacterial and archaeal; Figures 3A–D) and microeukaryotic (Figures 3E–H) datasets over the entire cohort. Non-parametric estimation of community richness for the individual time points according to Chao (1984) for prokaryotes and microeukaryotes showed comparable trends to the estimation of richness based on the numbers of different OTUs (Supplementary File 1, Figure S4). Given the sparseness and low abundance of archaeal OTUs detected by 16S rRNA gene amplicon sequencing, the observed patterns regarding prokaryotic diversity were mostly driven by bacterial taxa.

A significantly higher bacterial richness (number of different OTUs) was observed for the meconium samples compared to all other collection time points (Figure 3A, Supplementary File 1, and Table S3). In general, the inter-individual variability in richness was high on the first two sampling dates. The lowest richness of any sample was observed on day 3 *postpartum* and the overall median richness was lowest on day 5. The median richness increased subsequently and stabilized between day 28 and 150 (Figure 3A). The observed microeukaryotic richness tended toward a lower median richness at the end of the first year and showed a high level of variability throughout the first year of life (Figure 3E; Supplementary File 1, Table S4).

Shannon diversity and evenness metrics (Figures 3B,C, respectively) showed comparable trends for prokaryotic OTUs, i.e., a decrease in diversity and evenness with a concomitant decrease in variation in both diversity and evenness between individuals until day 5 *postpartum*. This was followed by a gradual increase for the subsequent collection time points. The observed microeukaryotic diversity and evenness (Figures 3F,G, respectively) followed no discernible trends compared to the bacterial data and exhibited constantly high levels of inter-individual variation. When linking samples according to the type of milk the infants received per time point, it became apparent that at day 5 and 28, infants that received combined feeding and formula-fed infants had a significantly lower microeukaryotic diversity compared to breast milk-fed infants (*P* = 0.01 at day 5 and *P* = 0.03 at day 28; Kruskal–Wallis test).

We calculated the Soerensen distance between the community structure at each time point and the community structure of the same individual in the most mature sample, i.e., usually the sample collected at 1 year, and compared the distances as a measure for maturity. For the prokaryotic dataset, the distances to the most mature sample exhibited a decreasing trend over time (Figure 3D). The observed patterns suggested a gradual development toward the 1 year samples, with day 150 exhibiting significantly more similarities to the most mature samples compared to the samples collected at day 1 (*P* = 0.009; Wilcoxon rank sum test). The same trend was observed for the Spearman correlation between the different time points (Supplementary File 1, Figure S5A), with samples of day 150 being significantly more correlated to the most mature microbiome than samples of day 1 (*P* = 0.004; Wilcoxon rank sum test). In contrast, the distances to the most mature microbial composition for the microeukaryotic microbiota (Figure 3H) as well as the Spearman correlation (Supplementary File 1, Figure S5B) displayed high variability among infants and between time points, and remained variable over time without reaching a certain level of maturity in regard to the 1 year samples.

### Comparison of microbiome community profiles of VD and CSD infants

Absolute quantification of 16S rRNA gene counts by qPCR showed that CSD infants carried significantly lower bacterial loads and thereby a decreased colonization density at day 3 and day 150 (*P* = 0.03 and *P* = 0.04 respectively; Figure 1A; Wilcoxon rank sum test). At the same time, CSD infants had microbial community structures with a significantly higher richness compared to VD infants at day 3 (*P* = 0.02; Wilcoxon rank sum test; Figure 3A).

**Figure 4:**
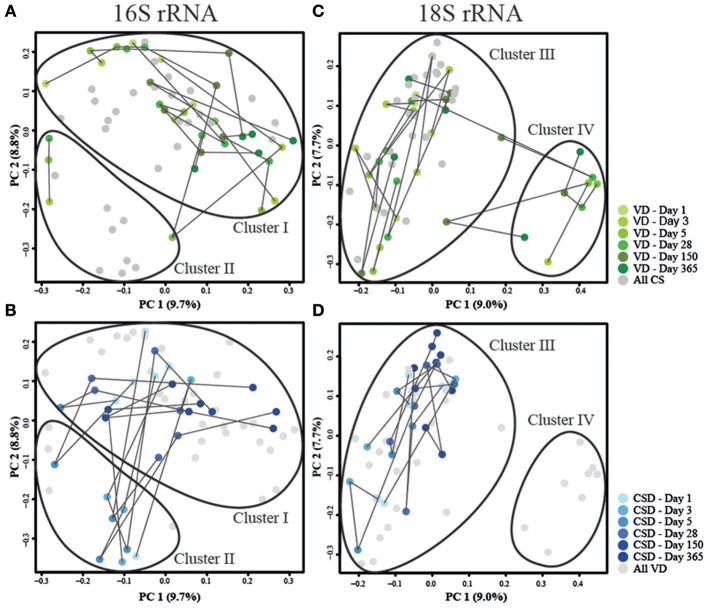
**Principal coordinate analyses of Jensen–Shannon distances for prokaryotic and microeukaryotic rRNA gene amplicon sequencing data**. Depiction of **(A,C)** data from VD infants in green and **(B,D)** CSD infants in blue for prokaryotes and microeukaryotes, respectively. Sampling time points are represented by shadings, with lighter colors depicting an earlier sampling time point. Lines connect samples which originated from the same infant according the order of sampling. Samples that are the focus of the corresponding other sub-panel are shaded in gray. Cluster delineations were added manually after computing the cluster membership of each sample using the partitioning around medoids (pam) function contained in the R package “cluster” (Maechler et al., 2016).

To provide an overview of the development of the microbiome of the eight VD (34 samples) and the seven CSD infants (30 samples), the 16S and 18S rRNA gene amplicon data were represented by an ordination of their respective Jensen–Shannon distances (Figure 4), a method that is commonly used for human microbial community structure analyses (Koren et al., 2013). Clusters on the PCoA plots were defined by partitioning around medoids (Maechler et al., 2016). For the prokaryotic community structure, samples collected at 1 year clustered together independently of delivery mode (Cluster I in Figures 4A,B), whereas most samples collected for CSD infants around days 3 and 5 *postpartum* were located in Cluster II (Figure 4B). In order to identify cluster-specific taxa, we compared the taxa in both clusters using DESeq2, resulting in 52 OTUs that were significantly different in their DESeq2-normalized read numbers between both clusters (Supplementary File 6). Among the top 10 OTUs with the smallest adjusted *P*-values ranging from 1.41^*^10^−18^ to 3.06^*^10^−04^, 6 OTUs belonged to the genus *Streptococcus* and always one OTU belonged to the genera *Proteus, Haemophilus*, and *Rothia*, which all exhibited increased abundances in Cluster II; and one OTU classified as *Bifidobacterium* spp. which was more abundant in Cluster I.

Similar to the 16S rRNA gene sequence data, the 18S rRNA data exhibited two clusters (Figures 4C,D). One cluster (Cluster III) comprised all samples except for the samples belonging to three VD infants (Cluster IV), while the microeukaryotic community composition of one VD infant transitioned between both clusters (Figure 4C). When comparing the taxonomic compositions in samples between both clusters (III and IV) using the Wilcoxon rank sum test and adjusting for multiple testing, eight OTUs, with six unclassified OTUs and two OTUs classified as *Candida* spp., were detected to be differentially abundant in both clusters with *P*-values ranging between 5.94^*^10^−10^ and 2.63^*^10^−02^ (Supplementary File 7). These OTUs were increased in their abundances in samples belonging to Cluster IV, but were most often missing or decreased in abundance in samples from Cluster III. Additionally, samples that fell into Cluster IV, were collected from vaginally delivered infants that were either undergoing weaning, were fed with formula milk or received a mixed combination of breast and formula milk but were not exclusively breast-fed at the given time point.

### Depletion of bacteroidetes in CSD infants

**Figure 5:**
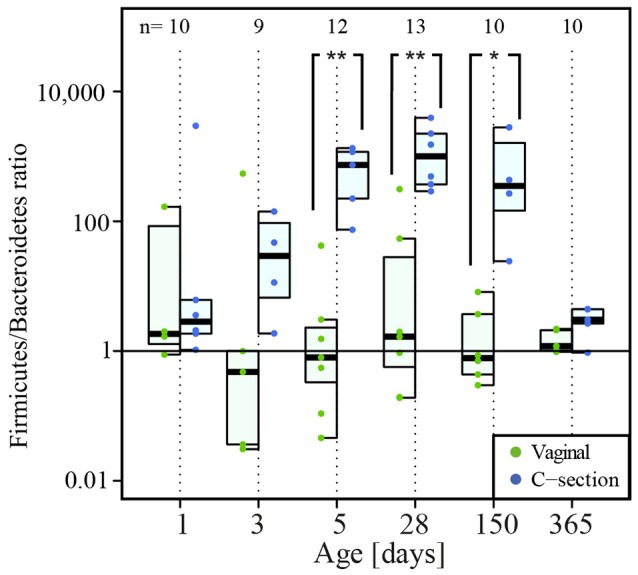
**Firmicutes/Bacteroidetes ratio over time**. The numbers of samples per collection time point are given at the top of the graph. Significant differences obtained by Wilcoxon rank sum test and according to delivery mode are represented by asterisks (^*^when *P* < 0.05; ^**^ when *P* < 0.01). CSD, C-section delivery; VD, vaginal delivery. Fecal samples originating from VD infants are represented on the left side of each barplot and by green points, samples from CSD infants are represented on the right side of each barplot and by blue points.

The most profound difference between CSD and VD infants was observed for the Firmicutes/Bacteroidetes ratio. While both phyla were approximately equally abundant in the VD infants (Figure 5), the corresponding ratio was significantly higher for CSD infants at days 5 (*P* = 0.006), 28 (*P* = 0.005), and 150 (*P* = 0.01; Wilcoxon rank sum test) while the proportional abundance for the phylum Bacteroidetes was significantly decreased in samples from CSD infants over most of the sampling time points (day 5: *P* = 0.006, day 28: *P* = 0.003, day 150: *P* = 0.01, day 365: *P* = 0.04; Wilcoxon rank sum test; Supplementary File 1, Figure S6A). At the same time, there was a concomitant increase in Firmicutes at day 5 in CSD infants (*P* = 0.01; Wilcoxon rank sum test). Preceding the drastic decrease in Bacteroidetes at day 5, there was already a significant difference at day 3 between infants born at different gestational ages, whereby full term (≥39 weeks) infants showed a higher relative abundance of Bacteroidetes when compared to late preterm (34–36 weeks) and early term (37–38 weeks) born infants (*P* = 0.05; Kruskal–Wallis test; Supplementary File 1, Figure S7).

**Figure 6:**
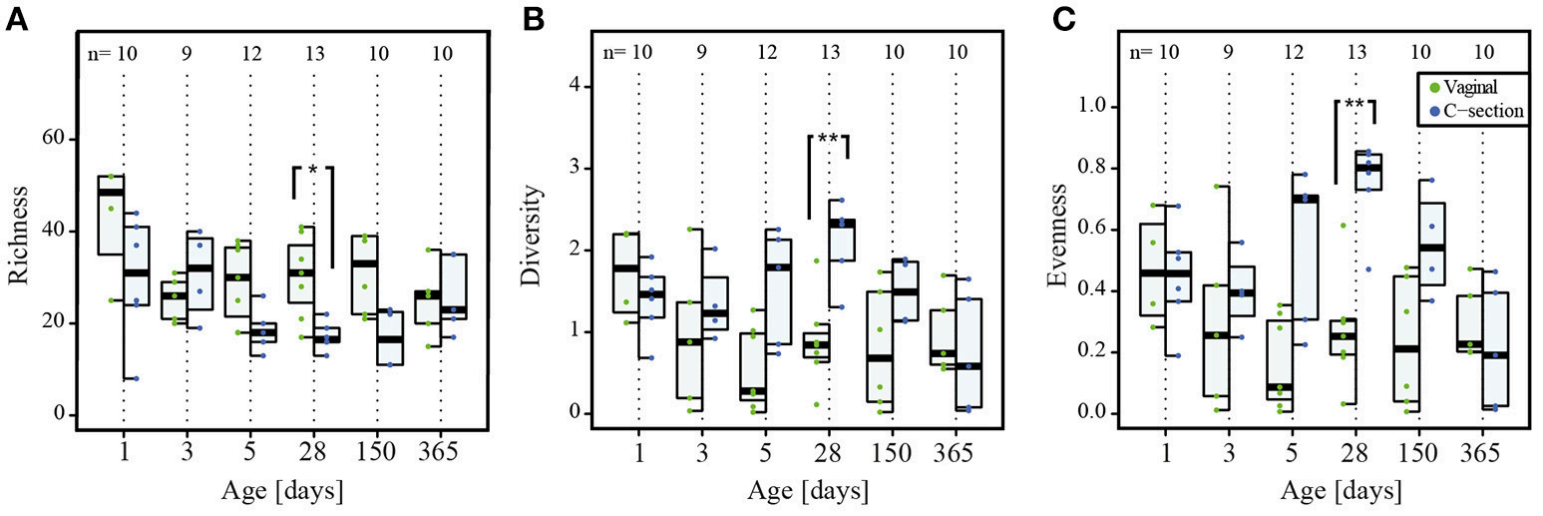
**Colonization by Bacteroidetes phylum**. Per collection time point depiction of **(A)** richness (number of OTUs), **(B)** diversity (Shannon's diversity index), and **(C)** evenness (Pielou's evenness index) of the phylum Bacteroidetes. The numbers of samples per collection time point are provided at the top of the graph. Significant differences as assessed by Wilcoxon rank sum test and according to delivery mode are represented by asterisks (^*^ when *P* < 0.05; ^**^ when *P* < 0.01). CSD, C-section delivery; VD, vaginal delivery. Fecal samples originating from VD infants are represented on the left side of each barplot and by green points, samples from CSD infants are represented on the right side of each barplot and by blue points.

In addition, we also more specifically analyzed richness, evenness, and diversity within the Bacteroidetes phylum (Figure 6). We observed a significant decrease in the Bacteroidetes richness in CSD infants at day 28 compared to VD infants (*P* = 0.01; Wilcoxon rank sum test; Figure 6A). The relative abundance of the genus *Bacteroides*, which made up more than 10% of the reads in most VD infants at days 28 and 150, exhibited a significant decrease in abundance associated with a delayed colonization in CSD infants (*P* = 0.04 at day 28 and 0.01 at day 150; Wilcoxon rank sum test; Supplementary File 1, Figure S6B). Due to this significant decrease in relative abundance of *Bacteroides* spp. compared to earlier and later time points in CSD infants and the subsequent shift in dominance inside the Bacteroidetes phylum, the diversity and evenness inside this phylum at day 28 were significantly increased (*P* = 0.005 for both; Wilcoxon rank sum test; Figures 6B,C). The different measures of diversity and evenness within the Firmicutes phylum did not show any significant differences between both delivery modes.

### Additional differences in prokaryotic community structure in CSD infants

We further aimed to determine whether other bacterial taxa also showed different changes in CSD infants compared to VD infants during the first year of life. We identified taxa that were differentially abundant according to delivery mode at each collection time point. After filtering the resulting 88 differentially abundant OTUs according to a cumulative read count above 10,000, we retrieved 29 OTUs with a positive fold change in CSD infants compared to VD infants and four OTUs that exhibited a negative fold change (Supplementary File 8). The same analysis was performed at the genus level and resulted in three genera with a negative fold change and 20 with a positive fold change in CSD compared to VD infants (Supplementary File 9).

The fecal microbiome of CSD infants was associated with increased proportional abundances of, amongst others, OTUs assigned to the genera *Haemophilus* spp., *Streptococcus* spp., *Enterobacter* spp., *Propionibacterium* spp., *Staphylococcus* spp., and the genus *Lactobacillus* over the first year of life. Furthermore, the microbiome of CSD infants contained lower proportions of *Bacteroides* spp. and *Parabacteroides* spp.

**Figure 7:**
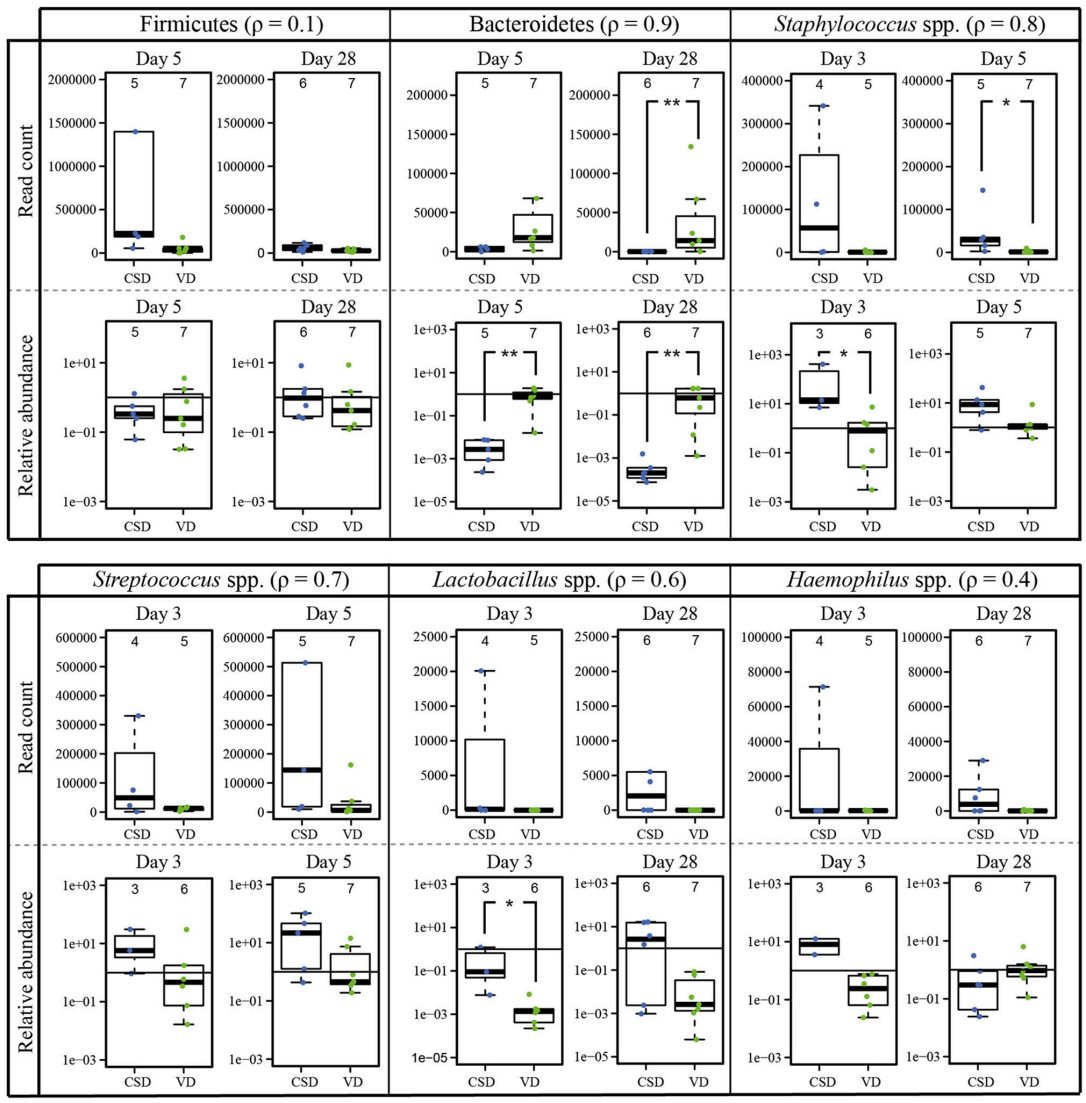
**qPCR validation of 16S rRNA gene sequencing data based differences according to delivery mode**. Comparison of the DESeq2-normalized 16S rRNA read numbers and relative abundances (given on log scale) measured by qPCR for two phyla and four genera that were found to be significantly different between birth modes. For each comparison the Spearman correlation coefficient (ρ) was calculated and figures next to the taxa. The numbers of samples per collection time point are given at the top of each barplot. Significant differences according to a Wilcoxon rank sum test for delivery mode are represented by asterisks (^*^ when *P* < 0.05; ^**^ when *P* < 0.01). CSD, C-section delivery; VD, vaginal delivery. Fecal samples originating from CSD infants are represented on the left side of each barplot and by blue points, samples from VD infants are on the right side of each barplot and by green points.

In order to validate that CSD infants harbored substantially different relative abundances of certain prokaryotic populations compared to VD infants at certain time points, we amplified specific target regions of the genera *Staphylococcus* spp. and *Streptococcus* spp. (at days 3 and 5), *Haemophilus* spp., and *Lactobacillus* spp. (at days 3 and 28) and the two phyla Firmicutes and Bacteroidetes (at days 5 and 28), to calculate their relative abundances. Validation by qPCR was done on samples that were collected on days on which the differences in relative abundances between both delivery modes were most pronounced. All targeted differences between CSD and VD children obtained in the previous differential analysis could be confirmed by qPCR analysis for the specific collection time points (Figure 7).

## Discussion

### Detection of prokaryotic and microeukaryotic communities in meconium

A number of recent studies indicate that meconium samples are not sterile but contain complex bacterial communities (Jiménez et al., 2008; Gosalbes et al., 2013; Ardissone et al., 2014). In this context, the previously accepted dogma of intrauterine sterility has been questioned. According to our results based on qPCR analyses as well as 16S and 18S rRNA gene amplicon sequencing, representatives of all three domains of life were present in meconium samples. Given that DNA yield out of meconium samples was limited (Figure 1), it might be possible that this microbial DNA might not be derived from the samples but may in fact represent contaminants of the reagents used for DNA extraction (Salter et al., 2014; Jervis-Bardy et al., 2015). However, according to simultaneously conducted analyses, even a 1,000-fold dilution of DNA extracted from an adult stool sample did not considerably change the taxonomic composition compared to the undiluted as well as the 10- to 100-fold diluted samples (Supplementary File 1, Figure S2B). From these results, we deduce that potential reagent contaminants did not have any significant impact on the overall community structure observed in our study. Moreover, the fact that we observed a significantly increased prokaryotic richness and diversity in meconium samples (Figures 3A,B) stood in stark contrast to the results from the dilution series, which revealed a decreased richness along with a stable diversity in the low-yield samples due to several taxa being diluted out of the adult stool sample during the 1,000-fold DNA dilution process (Supplementary File 1, Figure S2A). Additionally, the sequencing of all “DNA mock extracts” yielded very low coverage, while the detection of representatives of all three domains of life by qPCR could be considered negative as well. Taking these results into account, we suggest that the detection of taxa inside the meconium samples is not an artifact but has to be considered genuine. Whether the neonatal GIT was colonized prenatally or whether detected microbial populations were acquired perinatally could not be assessed in the context of our study.

The bacterial richness was significantly higher in meconium samples than at later time points. Samples from the first day were also highly diverse and the taxa were evenly distributed compared to subsequent collection time points, which suggests that these samples captured the potential early pioneering microbiota, most of which did not stably colonize the GIT thereafter. The richness decreased during the following days as the initial colonizers took hold in the GIT. Some of the taxa detected in the meconium samples may have been present in later samples but were not captured due to the masking by the dominant taxa. At day 1, the most abundant bacterial taxa in all infants were *Escherichia/Shigella* spp., *Bifidobacterium* spp., *Enterobacter* spp., *Staphylococcus* spp., *Streptococcus* spp., *Prevotella* spp., and *Veillonella* spp., which have all been previously described in meconium samples as being pioneering genera of the human GIT (Gosalbes et al., 2013; Ardissone et al., 2014; Hansen et al., 2015). The latter four are either present predominantly on skin (Dominguez-Bello et al., 2010), in colostrum or are typical inhabitants of the oral cavity (Cabrera-Rubio et al., 2012). Pioneering bacterial colonizers of the microbiome are usually facultative anaerobes, such as *Escherichia* spp. (Jiménez et al., 2008), as also observed in our study. These pioneers shape the gastrointestinal microbiome environment, promoting the subsequent colonization by strict anaerobes such as *Bacteroides* spp., *Clostridium* spp., and *Bifidobacterium* spp., which were already detected in samples collected on day 1 in our study. Overall, the earliest bacterial colonizers detected in all meconium samples included both facultative and strict anaerobic taxa suggesting that the GIT rapidly transitions toward an anaerobic environment after birth. *Bifidobacterium* spp., which was the taxon with the highest read counts across all samples, are important for neonatal health and are known to have beneficial effects for the host through their breakdown of dietary carbohydrates, the products of which directly feed into host metabolism (Davis et al., 2011). *Bifidobacterium* spp. are colonizers of the vaginal microbiome and are supposedly transferred to the infant during vaginal delivery (Dominguez-Bello et al., 2010). However, while in line with previous findings (Jakobsson et al., 2014), no significant difference in *Bifidobacterium* spp. abundances between VD and CSD infants could be detected for meconium samples, suggesting that other routes of transmission are also very likely during neonatal colonization. Additionally, the growth of this specific taxon is promoted selectively by prebiotic oligosaccharides present in the maternal colostrum and breast milk (Zivkovic et al., 2011; Yu et al., 2013).

Results from the quantitative real-time PCR assay suggested that archaea, even if low in abundance, were amongst the earliest colonizers of the neonatal GIT microbiome. The only methanogenic archaeon that was identified using the 16S rRNA gene amplicon sequencing was *Methanosphaera* spp., which was exclusively detected in VD infant 5 at day 1. This human archaeal commensal has a highly restricted energy metabolism (Fricke et al., 2006), which makes it a specialized member of the gastrointestinal microbiome. Archaea have been shown to be ubiquitous members of the adult GIT microbiome (Dridi et al., 2009), were sporadically detected in the vaginal environment (Belay et al., 1990), and were shown to colonize the skin surface (Probst et al., 2013) and the oral cavity (Nguyen-Hieu et al., 2013). As the presence of archaea was also apparent in CSD infants and also in samples collected at day 1 in our study, we can postulate that transmission paths besides vaginal transmission, such as fecal-oral, oral-oral, or by skin contact most probably occur perinatally.

The earliest microeukaryotic colonizers included *Exobasidiomycetes* spp. and 2 OTUs classified as *Saccharomyces* spp., which were detected in meconium from CSD infants, whereas *Dothideomycetes* spp. and *Pezizomycotina* were detected mostly in VD infants. A recent study found *Saccharomyces* spp. and *Dothideomycetes* spp. to be present in more than half of the analyzed adult stool samples (Mar Rodríguez et al., 2015), which make them common taxa of the human GIT microbiome. As the vaginal tract is largely colonized by yeasts such as *Saccharomyces* spp., vaginal delivery is supposedly linked to neonatal colonization by yeasts through vertical transmission from the mother's vaginal microbiome or through horizontal transmission from the environment and hands of family members as well as health care workers (Lupetti et al., 2002; Bliss et al., 2008).

If pioneering microbiota, including representatives from all three domains of life, have the potential to colonize the GIT microbiome prenatally (Greenhalgh et al., 2016), according to our results, birth still marked the time point of extensive microbial colonization, which further defined microbial succession. Clearly, more work needs to be undertaken on meconium and the crucial first hours of life to ascertain the different sources of the pioneering microbiota.

### Colonization and succession within the neonatal GIT microbiome by prokaryotes and microeukaryotes during the first year of life

The progressive nature of neonatal GIT colonization and succession by prokaryotes was apparent through an increase in absolute prokaryotic DNA load (Figure 1), overall alterations to community compositions (Figure 2) as well as changes in richness, diversity and evenness (Figure 3). A general trend regarding the prokaryotic community members is that their structure matures over the course of the first year of life. This maturation was reflected by increases in diversity and evenness over time and shortly after an initial decrease from day 1 to 5 after birth. The fact that diversity and evenness keep increasing over time has already been reported in previous studies (Yatsunenko et al., 2012; Jakobsson et al., 2014). However, in our study, significant differences in diversity and evenness between subsequently sampled time points were observed as early as between days 5 and 28 (Figures 3B,C). The prokaryotic richness stabilized between days 28 and 150 (Figure 3A). Similarly, the dissimilarity index, reflecting the distance of the taxonomic composition of each sample to the last collected sample per child, showed a decreasing trend (Figure 3D), highlighting that the microbiome composition gradually changed from a neonatal profile toward the most mature composition available by 1 year of age.

A previous study, focusing on neonatal colonization, has found archaea to be transiently and almost exclusively present in the first few weeks of life during their sample collection, which was conducted until around 17 months (Palmer et al., 2007), whereas archaea are considered core members of the adult GIT microbiome (Dridi et al., 2009). While archaea could not be identified confidently by amplicon sequencing in our study after the first day, the qPCR assays using an archaea-specific primer set suggested that they were indeed present in 90% of all samples, opposing previous results and highlighting their potential importance in the maintenance of inter-species community networks (Hansen et al., 2011). Although the 16S rRNA gene amplifying primer used for sequencing covered both domains bacteria and archaea, the nature of GIT microbiome profiles, with bacteria making up the large majority of the composition, likely caused a lack of primer availability for archaea, potentially explaining why this domain was more extensively detected with qPCR using the archaea-specific primers rather than using the more generic 16S rRNA gene primers used for the amplicon sequencing. In the future, dedicated archaeal and bacterial primer sets may be used to allow better resolution of the archaea.

When considering the microeukaryotic community, no clear successional patterns were discernible. In line with previous studies involving culture-independent analyses of the GIT microbiome, most detected fungal taxa belonged to the phyla Ascomycota and Basidiomycota (Ott et al., 2008; Scanlan and Marchesi, 2008). In contrast to previous reports on adult GIT microbiota (Scanlan and Marchesi, 2008), identities and abundances of detected microeukaryotic taxa fluctuated strongly throughout the first year of life. Similarly, richness, diversity and evenness indices did not follow discernible trends over time (Figures 3E–G). However, we found a more rapid microeukaryotic diversification in infants who were fed exclusively breast milk between days 5 and 28 as well as a separation of samples on the PCoA plot that were collected from vaginally delivered infants either undergoing weaning, that were fed with formula milk or that received a mixed combination of breast and formula milk but were not exclusively breast-fed at that time point (Figures 4C,D). These findings suggest a possible link between the infants' feeding regimes and early changes to microeukaryotic community development in the human GIT. When considering the intra-individual dissimilarity index in addition to the apparent large inter-individual variation, our findings indicated that the microeukaryotic community members were more dynamic compared to their prokaryotic counterparts (Figure 3H). A previous study in the mouse GIT observed similar results with fungal populations varying substantially, while bacterial populations remained relatively stable over time (Dollive et al., 2013). Typically, only a small number of common genera, such as the genus *Saccharomyces*, and a large number of spurious taxa that have been barely reported previously have been described to form part of the human GIT microbiome (Suhr and Hallen-Adams, 2015). The specific characteristics of these rare taxa suggest that they do not persist inside the GIT microbiome but are likely more transient in nature when compared to bacteria (Suhr and Hallen-Adams, 2015). Also, fewer microeukaryotic species and individual microeukaryotes are found in the human GIT than bacteria, potentially explaining why the microeukaryotic community may be less robust in comparison to bacteria (Underhill and Iliev, 2014). Furthermore, according to our results, the general lack in successional patterns with regards to the microeukaryotes suggested that either the neonatal GIT would not allow any durable colonization by microeukaryotes, including known common microbiome members such as *Blastocystis* spp. or *Dientamoeba fragilis* (Scanlan et al., 2014), that the required ecological niches did not exist in the GIT during the first year of life or that those microeukaryotes never actually stably colonize the GIT as suggested before by Suhr and Hallen-Adams (2015).

### Prokaryotic differences in colonization and succession between CSD and VD infants

Diversity and evenness measures were not significantly different between CSD and VD infants (Figures 3B,C), in contrast to the results from another recent study (Jakobsson et al., 2014). However, a difference between VD and CSD infants was observed early on in terms of the prokaryotic richness, which was significantly increased in CSD infants (Figure 3A). This finding could reflect the different pioneering taxa between both delivery groups. Furthermore, we found that generally lower amounts of DNA were extracted from stool of CSD infants compared to VD infants using the same extraction protocol, suggesting a delay in the acquisition of prokaryotic biomass in the GIT of CSD infants. While the DNA yields quickly increased over time for VD infants, CSD infants showed a slower acquisition of a similar colonization density, which could be explained by either a delay in exposure to bacteria or the inoculation by fundamentally different microbial taxa, which could be less adapted to the human GIT and therefore exhibited lower growth rates.

In addition to differences in microbial loads during the first days after birth (Figure 1A), we identified apparent differences in early prokaryotic succession. For instance, several samples taken from CSD infants during days 3 and 5 were found to share similarities in community structure (Cluster II) that were not typically observed in samples from VD children (Figures 4A,B). These similarities included increased relative abundances of *Streptococcus* spp. and *Staphylococcus* spp. (Supplementary File 6). These taxa are typically found in the oral cavity and on the skin surface and are supposedly transferred from mother to infant through skin contact in CSD infants (Dominguez-Bello et al., 2010). Furthermore, these samples showed significantly decreased relative abundances of *Bacteroides* spp. and *Bifidobacterium* spp., whose colonization has been shown to be delayed in CSD infants (Adlerberth et al., 2006; Penders et al., 2006; Sufang et al., 2007; Biasucci et al., 2008; Dominguez-Bello et al., 2010). Interestingly, allergic diseases have been previously associated with a low prevalence of *Bacteroides* spp. and *Bifidobacterium* spp. (Björkstén et al., 1999; Watanabe et al., 2003), and low levels of *Bifidobacterium* spp. together with significantly increased levels of *Staphylococcus* spp. have been associated with childhood obesity (Kalliomäki et al., 2008). Generally, the genus *Bifidobacterium* is associated with an enhanced epithelial barrier function (Cani et al., 2009). These findings are in line with the statistically higher risks of CSD infants of developing obesity (Mueller et al., 2015) or allergic diseases (Abrahamsson et al., 2012, 2014). Although the differences observed in our study were compelling, whether the observed microbiome signatures in CSD infants are directly causally linked to disease development later in life has yet to be established in larger infant cohorts with longer-term follow-up. After day 150, the observed differences between CSD and VD infants became less pronounced. This observed trend could have been driven by weaning the infants from an exclusive milk diet and/or the introduction of solid food around the same time. Previous studies showed that through the introduction of new and diverse nutrients, the microbiome quickly changes toward a more adult-like profile, thereby decreasing early differences in profiles caused by delivery mode or other maternal and neonatal characteristics (Fallani et al., 2011; Koenig et al., 2011).

Although the delivery mode appeared to have the strongest influence on differences between the infants, other factors may also contribute to the observed patterns. Most notably, reduced gestational age, higher maternal age, a higher maternal BMI, and specific maternal antibiotic treatments are commonly observed in the context of CSD (van Schalkwyk et al., 2010; Al-Kubaisy et al., 2014; Delnord et al., 2014; Klemetti et al., 2016). For example, gestational age may have been an additional factor driving the early Bacteroidetes depletion. Already at day 3, Bacteroidetes were significantly decreased in five infants that were born late preterm (34–36 weeks) or early term (37–38 weeks) compared to four full term infants (≥39 weeks; Supplementary File 1, Figure S7). Known effects of preterm delivery on neonatal microbiome colonization include reduced levels of strict anaerobes such as *Bifidobacterium* spp. and *Bacteroides* spp. (Arboleya et al., 2012, 2016) and a slower microbial succession (La Rosa et al., 2014), all of which were observed in our study for samples collected from CSD infants. Another factor may have been maternal perinatal antibiotics intake which was associated with significantly lower amount of prokayotic DNA at day 5 (and a similar trend at days 1 and 28; Supplementary File 1, Figure S1). Importantly, the antibiotic intake of the mother may have effects on the GIT microbiome of the infant, either directly, e.g., transfer from maternal blood via the blood-placental barrier prior to birth (Pacifici, 2006), or indirectly, e.g., transfer of antibiotics via breast milk *postpartum* (Zhang et al., 1997). As antibiotic administration is recommended in case of delivery by C-section, this could be yet another factor that had a negative influence on the observed delay in colonization and succession in CSD infants while even potentially inhibiting the succession rate in VD infants to a certain extent. As the present study cohort was limited in size, the obtained data cannot be considered representative of a more general situation such that other environmental factors may have non-negligent influences on the composition of the developing microbiome as well.

Besides shifts in the early successional patterns and factors that could enhance the observed delay in colonization, we also observed fundamental differences in the taxonomic composition of CSD infants compared to VD infants and over all time points, such as a significantly decreased relative abundance of Bacteroidetes (Supplementary File 1, Figure S6A), which remained prominent even at 1 year. The most drastic difference in microbiome composition was an elevated Firmicutes/Bacteroidetes ratio observed in CSD infants between days 5 and 150 (Figure 5). An elevated Firmicutes/Bacteroidetes ratio has been previously linked to an increased energy harvesting capacity by the host and its potential contribution to the development of metabolic disorders such as diabetes, obesity, or metabolic syndrome in adulthood (Turnbaugh et al., 2006), although more recent findings seem to suggest that evidence for the implication of the Firmicutes/Bacteroidetes ratio in human health may be weaker than previously assumed (Sze and Schloss, 2016). The differential analysis detected statistically significant alterations of additional bacterial taxa in CSD infants over all time points, of which several were also validated by qPCR (Figure 7, Supplementary Files 8, 9). As already highlighted previously, CSD infants harbored lower proportions of *Bacteroides* spp. and *Parabacteroides* spp., which again point out that CSD infants were subject to a delayed rate of colonization for the phylum Bacteroidetes and more specifically the genera *Bacteroides* and *Parabacteroides*. Taxa commonly derived from skin, the oral cavity and the environment exhibited an enrichment in CSD infants. These taxa included *Haemophilus* spp., *Streptococcus* spp., *Enterobacter* spp., *Propionibacterium* spp., and *Staphylococcus* spp., which have been previously found to be enriched in CSD infants, supposedly through skin microbiome transfer from mother to the newborn after birth (Dominguez-Bello et al., 2010; Bäckhed et al., 2015). Interestingly, CSD infants in our study were also enriched in the genus *Lactobacillus*. As *Lactobacillus* spp. are usually dominant in the vaginal microbiome, they are supposedly transferred from mother to infant during vaginal delivery, thereby being deficient and delayed in CSD infants (Grönlund et al., 1999; Adlerberth et al., 2006; Dominguez-Bello et al., 2010; Rutayisire et al., 2016). Other routes of colonization however also include the administration of breast milk (Bäckhed et al., 2015).

### General delay in colonization rates in CSD infants

Overall, archaea and fungi were more often detected by qPCR in VD infants compared to CSD infants, and the yield of fungal DNA was lower in CSD infants compared to VD infants, except at 1 year and after introduction of solid food in all infants. These findings indicate that the previously described delay in colonization and succession observed for bacteria in CSD infants may actually affect all three domains of life, adding valuable information to our current knowledge regarding neonatal colonization of the GIT microbiome.

The initial microbiome colonization process is especially crucial for the early stimulation and maturation of the immune system (Rizzetto et al., 2014; Houghteling and Walker, 2015; Mueller et al., 2015), such that the observed delay of all three domains of life in CSD infants may result in an altered immunostimulatory effect, which in turn may potentially have long-lasting effects in relation to human health. Whether the early disturbance and delay of the colonization and succession processes in CSD infants could potentially exacerbate or contribute to the higher risk of CSD infants to develop certain diseases, therefore requires additional immunological data. However, what has been observed so far is that due to the close contact between the developing GIT, the underlying immune system and the colonizing bacteria, the early microbiome acts as an important interface in the neonatal development of the immune system (Björkstén, 2004; Caicedo et al., 2005; Rautava and Walker, 2007; Eberl and Lochner, 2009). Substantial shifts of neonatal taxonomic compositions or disruptions of natural colonization and succession processes may thereby lead to changes in the long-term developmental processes and subsequent altering of immune development. Additionally, the timing of colonization plays an important role in neonatal immune programming. Previous studies on mouse models observed that a delayed microbial GIT colonization of germ-free mice caused long-term changes in the immune system (Sudo et al., 1997; Rautava and Walker, 2007; Eberl and Lochner, 2009; Hansen et al., 2012; Olszak et al., 2012). These recent findings suggest that the composition and timing of early neonatal colonization in CSD infants are important factors influencing the microbial education of the developing immune system, which could result in long-term persistent alterations in systemic gene expression and increased disease predispositions.

## Conclusions

Here, we describe for the first time the colonization of the neonatal human GIT resolved to all three domains of life. We demonstrate that bacteria but also archaea and microeukaryotes, predominantly fungi, are detectable in meconium samples and are thereby among the earliest colonizers of the neonatal GIT microbiome.

In contrast to the patterns observed for prokaryotes, microeukaryotic abundances fluctuated strongly over time, suggesting that the microeukaryotic community did not reach a stable colonization state during the first year of life. Based on our results, the milk-feeding regime appeared to impact the early microeukaryotic colonization and diversification process. An important question in this context is whether a diverse microeukaryotic microbiome is more resilient to disturbances and beneficial for the host as it has been proposed for bacterial constituents of the GIT microbiome.

As for the differences in colonization and succession between VD and CSD infants during the first year of life, our findings highlight that CSD infants experience a delay in colonization and succession affecting all three domains of life, generally complementing and further extending previous observations. Substantial shifts in the community compositions started as early as day 5 and were potentially caused by differences in time of incidental exposure to bacteria from the environment in CSD infants. We further suggest a potential link to earlier gestational age and maternal antibiotics intake. Given that the early microbiome supposedly shapes the immune system, our observations that CSD infants exhibited a different succession pattern early on raises the possibility that disturbances to the microbiome in the early stages of neonatal development might have long-lasting health effects. Although major differences between VD and CSD infants were less apparent at 1 year of age, the question whether differences in the early stimulation of the immune system by either the VD or the CSD microbiomes may change the infants' response to later perturbations, such as during the introduction of solid food, will require further in-depth studies. In order to answer these questions, high-frequency sampling of GIT microbiota along with resolving crucial immune characteristics over longer periods of time should be undertaken.

Further additional work is required to determine at which stage of infant development the GIT microbiome acquires a mature archaeal community, as well as when the transition between the highly dynamic early microeukaryotic microbiota and the stable adult microeukaryotic community occurs. Open questions in this context revolve around which role these two domains play with respect to neonatal host metabolism, how they influence the host's immune system and how they influence the GIT microbiome through providing specific metabolic functions.

Our findings provide an important account of the neonatal colonization and succession within the human GIT microbiome by bacteria, archaea, and microeukayotes. In particular, our findings highlight the need for studying all three domains of life in future longitudinal studies of microbial colonization and succession within the human GIT to finally understand how the individual taxa affect host physiology and how differences in colonization and succession of all three domains may contribute to the development of diseases later in life.

## Author contributions

LW carried out the qPCR assays, data processing, comparative analyses of the 16S and 18S rRNA gene amplicon sequencing data and data interpretation, participated in the biomolecular extractions and wrote the manuscript. AHB, AH, CL, Cd, and PW were involved in data analysis and interpretation. AHB contributed toward biomolecular extractions and writing the manuscript. AH participated in the design of the study and in the sample and data collection. EM, SN, and CL participated in data analysis and critical revision of the manuscript. LH and AA participated in the data processing of the 18S rRNA gene amplicon sequencing data and provided important advice. LB and JB participated in the sample collection and processing. Cd and PW conceived and coordinated the study and participated in its design and in writing the manuscript. All authors read and approved the final manuscript.

## Funding

The present work was partially funded by the Integrated BioBank of Luxembourg. Additional support was provided by an ATTRACT program grant (ATTRACT/A09/03), a CORE programme grant (CORE/15/BM/10404093) and a European Union Joint Programme in Neurodegenerative Disease research grant (INTER/JPND/12/01) to PW as well as Aide à la Formation Recherche grants to LW (AFR PHD-2013-5824125), CL (AFR PHD/4964712), and SN (AFR PHD-2014-1/7934898), a CORE junior to EM (C15/SR/10404839) all funded by the Luxembourg National Research Fund (FNR). The continuation of the study has been ensured with the generous financial support of the Fondation André et Henriette Losch. AA was funded by a grant from the Swedish Research Council VR (grant 2011-5689). The funding bodies had no impact on the design of the study, data collection, analysis, interpretation of data, nor in writing the manuscript.

### Conflict of interest statement

The authors declare that the research was conducted in the absence of any commercial or financial relationships that could be construed as a potential conflict of interest.

## Footnotes

^1^http://www.bioinformatics.babraham.ac.uk/projects/fastqc/

^2^https://github.com/EnvGen/Tutorials/blob/master/amplicons-no_overlap.rst

^3^https://cran.r-project.org/web/packages/vegan/index.html

## Supplementary material

Figure S1 **Impact of maternal antibiotic intake prior to birth on the yield of prokaryotic DNA from neonatal stool samples**.

Figure S2 **Analysis of 16S rRNA gene amplicon data from a DNA dilution series**.

Figure S3 **Quality of the archaeal sequencing reads**.

Figure S4 **Microbial richness during colonization and succession of the infant GIT**.

Figure S5 **Spearman correlations between samples from each time point compared to the individual most mature microbial community profiles represented by samples collected at the final time point per infant**.

Figure S6 **Differences between delivery modes in relation to relative abundances of Bacteroidetes and *Bacteroides spp***.

Figure S7 **Relative abundances of Bacteroidetes in children born at different gestational ages**.

Table S1 **Milk feeding regime of the infants prior to the different sample collection time points**.

Table S2 **Results of Wilcoxon rank sum test comparing the yields measured for the prokaryotic and fungal DNA at different collection time points**.

Table S3 **Results of Wilcoxon rank sum tests for prokaryotic diversity, evenness, richness, and dissimilarity indices at different collection time points**.

Table S4 **Results of Wilcoxon rank sum tests for microeukaryotic diversity, evenness, richness, and dissimilarity indices at different collection time points**.

Supplementary File 1 **Figures S1–S7 and Tables S1–S4**.

Supplementary File 2 **Number of reads obtained from the 16S rRNA gene amplicon sequencing data at OTU level and in all samples**.

Supplementary File 3 **Number of reads obtained from the 18S rRNA gene amplicon sequencing data at OTU level and in all samples**.

Supplementary File 4 **Sequencing reads obtained from the 16S rRNA gene amplicon sequencing data at OTU level in meconium samples**.

Supplementary File 5 **Sequencing reads obtained from the 18S rRNA gene amplicon sequencing data at OTU level in meconium samples**.

Supplementary File 6 **List of OTUs with significantly different relative abundances between Clusters I and II**.

Supplementary File 7 **List of OTUs with significantly different relative abundances between Clusters III and IV**.

Supplementary File 8 **List of OTUs resulting from the differential analysis (DESeq2) that were significantly differentially abundant across all collection time points and comparing VD and CSD infants**.

Supplementary File 9 **List of genera resulting from the differential analysis (DESeq2) that were significantly differentially abundant across all collection time points and comparing VD and CSD infants**.

## Abbreviations

BMI: body mass index
Cp: Crossing point
CSD: cesarean section delivery
C-section: cesarean section
FNR: Luxembourg National Research Fund
CART-GIGA: Center of Analytical Research and Technology—Groupe Interdisciplinaire de Génoprotéomique Appliquée
GIT: gastrointestinal tract
IBBL: Integrated BioBank of Luxembourg
ISBER: International Society for Biological and Environmental Repositories
OTU: operational taxonomic units
pam: partitioning around medoids
PCoA: Principal Coordinate Analysis
qPCR: quantitative real-time PCR
RDP: Ribosomal Database Project
VD: vaginally delivered.
