# Supplementary material for: Colonization and Succession within the Human Gut Microbiome by Archaea, Bacteria, and Microeukaryotes during the First Year of Life
Source: Front Microbiol. 2017 May 2;8:738. doi: 10.3389/fmicb.2017.00738 (PMC5411419; doi:10.3389/fmicb.2017.00738)
Supplement: Figure S1 — Impact of maternal antibiotic intake prior to birth on the yield of prokaryotic DNA from neonatal stool samples. [file DataSheet1.docx]

**Supplementary Material**

**Supplementary Figures and Tables**

# Colonization and succession within the human gut microbiome by archaea, bacteria and microeukaryotes during the first year of life

Linda Wampach^1^, Anna Heintz-Buschart^1^, Angela Hogan^2^, Emilie E.L. Muller^1+^, Shaman Narayanasamy^1^, Cedric C. Laczny^1^°, Luisa W. Hugerth^3^, Lutz Bindl^4^, Jean Bottu^4^, Anders F. Andersson^3^, Carine de Beaufort^1,4^ and Paul Wilmes^1^*

**Author affiliations:**

1. University of Luxembourg, Luxembourg Centre for Systems Biomedicine, Esch-sur-Alzette, Luxembourg

linda.wampach@uni.lu,

anna.buschart@uni.lu

emilie.muller@unistra.fr

shaman.narayanasamy@uni.lu

cedric.laczny@ccb.uni-saarland.de

carine.debeaufort@uni.lu

paul.wilmes@uni.lu

^+^ Current affiliation:

° Current affiliation: Chair for Clinical Bioinformatics, Saarland University, Building E2.1, 66123 Saarbrücken, Germany

2. Integrated BioBank of Luxembourg, Luxembourg, Luxembourg

Angela.Hogan@ibbl.lu

3. KTH Royal Institute of Technology, Science for Life Laboratory, School of Biotechnology, Division of Gene Technology, Stockholm, Sweden

luisa.hugerth@scilifelab.se

anders.andersson@scilifelab.se

4. Centre Hospitalier de Luxembourg, Luxembourg, Luxembourg

Bindl.Lutz@chl.lu

Bottu.Jean@chl.lu

debeaufort.carine@chl.lu

* for correspondence: paul.wilmes@uni.lu

Tel. +352 46 66 44 6188

Fax +352 46 66 44 6949

**List of Supplementary Figures**

**Fig. S1.** Impact of maternal antibiotic intake prior to birth on the yield of prokaryotic DNA from neonatal stool samples.

**Fig. S2.** Analysis of 16S rRNA gene amplicon data from a DNA dilution series.

**Fig. S3.** Quality of the archaeal sequencing reads.

**Fig. S4.** Microbial richness during colonization and succession of the infant GIT.

**Fig. S5.** Spearman correlations etween samples from each time point compared to the individual most mature microbial community profiles represented by samples collected at the final time point per infant.

**Fig. S6.** Differences between delivery modes in relation to relative abundances of Bacteroidetes and *Bacteroides* spp..

**Fig. S7.** Relative abundances of Bacteroidetes in children born at different gestational ages.

**Fig. S1. Impact of maternal antibiotic intake prior to birth on the yield of prokaryotic DNA from neonatal stool samples.** Absolute quantification of yields of prokaryotic DNA (ng DNA/mg of stool) measured by quantitative real-time PCR over the course of the first year of life. The numbers of samples per collection time point are provided at the top of the graph. For the purpose of clarity, only significant differences obtained by Wilcoxon rank sum test between defined groups are indicated in the figure (* when < 0.05). Fecal samples originating from infants whose mothers did not receive any antibiotics prior to birth are represented on the left side of each subdivision (“No”), samples from infants whose mothers were administered antibiotics prior to birth are situated on the right side of each subdivision (“Yes”). CSD: C-section delivery, VD: vaginal delivery. Fecal samples originating from VD infants are represented by green points, samples from CSD infants by blue points.

**Fig. S2. Analysis of 16S rRNA gene amplicon data from a DNA dilution series.** (**A**) Calculated indices of diversity (Shannon), evenness (Pielou) and dissimilarity (Soerensen), and measured richness of a 16S amplicon sequenced DNA sample from adult stool and corresponding 10-, 100- and 1,000-fold dilutions. (**B**) Barplot of relative abundances of all 36 aggregated OTUs per sample. Sequences were classified to the highest taxonomic level that could be confidently assigned. Aggregated OTUs are color-coded according to the phylum they belong to.


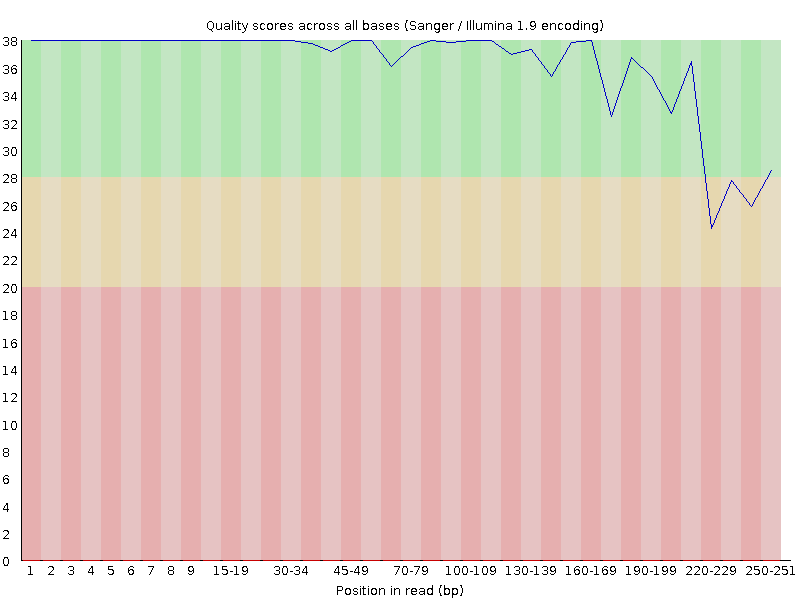

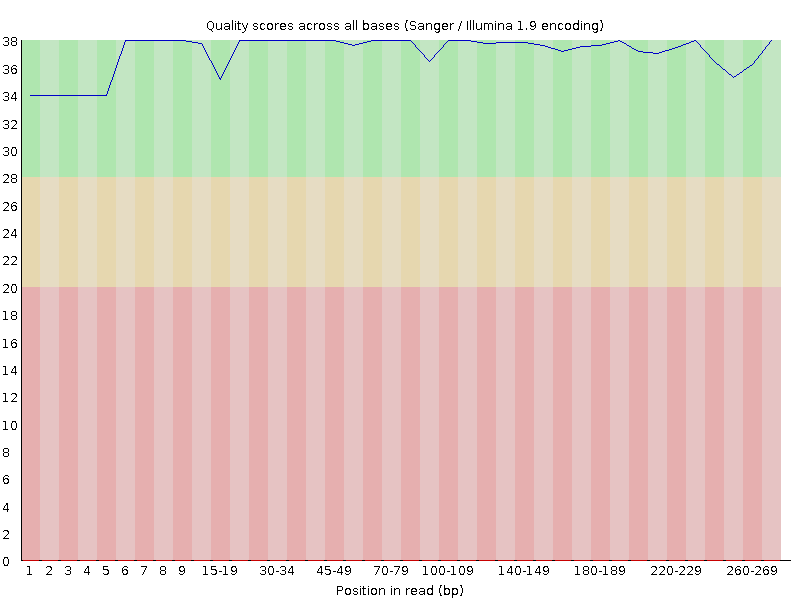
  **A** **B**

OTU 1,128


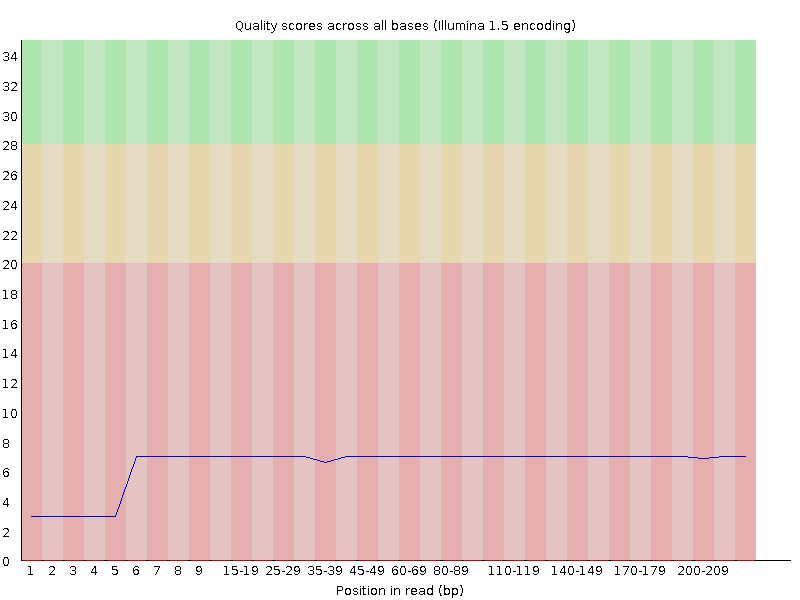

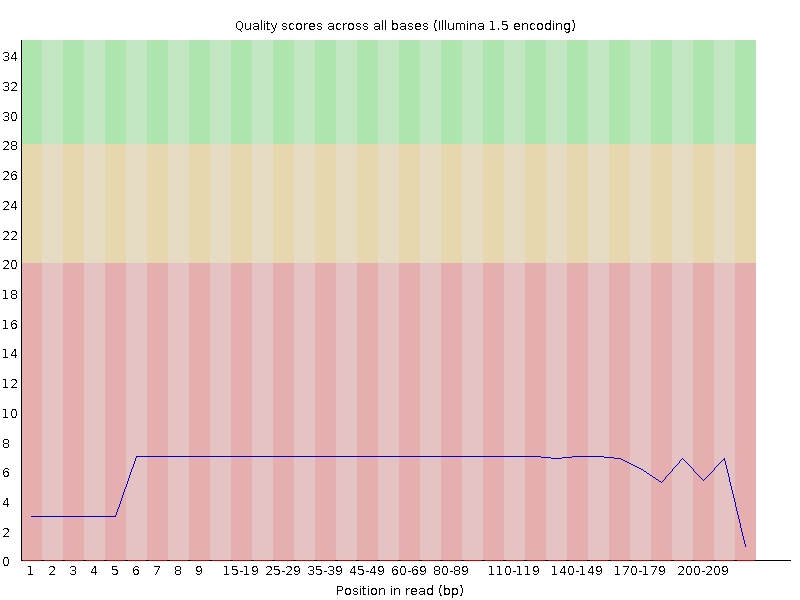
 **C**  **D**

OTU 693

**Fig. S3. Quality of the archaeal sequencing reads.** Overall quality measured for (**A**) forward reads and (**B**) reverse reads of OTU 1,128 (*Methanosphaera* sp.), and for (**C**) forward reads and (**D**) reverse reads of OTU 693 (*Methanobrevibacter* sp.). The read mappings were visualized using FASTQC.

**C**

| 16S | Day 1 | Day 3 | Day 5 | Day 28 | Day 150 | Day 365 |
| --- | --- | --- | --- | --- | --- | --- |
| Day 1 |  | 1.6E-02 | 1.7E-03 | 9.2E-03 | 1.7E-02 | 1.3E-02 |
| Day 3 |  |  |  |  |  |  |
| Day 5 |  |  |  |  | 3.4E-02 |  |
| Day 28 |  |  |  |  |  |  |
| Day 150 |  |  |  |  |  |  |
| Day 365 |  |  |  |  |  |  |
|  |  |  |  |  |  |  |
| 18S | Day 1 | Day 3 | Day 5 | Day 28 | Day 150 | Day 365 |
| Day 1 |  |  |  |  |  |  |
| Day 3 |  |  |  |  |  |  |
| Day 5 |  |  |  |  |  |  |
| Day 28 |  |  |  |  |  | 5.8E-03 |
| Day 150 |  |  |  |  |  |  |
| Day 365 |  |  |  |  |  |  |

**Fig. S4. Microbial richness during colonization and succession of the infant GIT.** (**A**) Non-parametric estimation of minimum community richness according to Chao *et al*. for the prokaryotic and (**B**) microeukaryotic data sets. The numbers of samples per collection time point are provided at the top of the graph. In A and B, Significant differences obtained by Wilcoxon rank sum test between consecutive time points are represented by asterisks (* when < 0.05; ** when <0.01). (**A**) & (**B**) Fecal samples originating from VD infants are represented on the left side of each barplot and by green points, samples from CSD infants are represented on the right side of each barplot and by blue points. CSD: C-section delivery, VD: vaginal delivery. (**C**) Information on all significant differences between collection time points for both data sets.

**Fig. S5.** **Spearman correlations between samples from each time point compared to the individual most mature microbial community profiles represented by samples collected at the final time point per infant.** Spearman correlation for (**A**) prokaryotic and (**B**) microeukaryotic community structures. The numbers of samples per collection time point are provided at the top of the graph. Significant differences obtained by Wilcoxon rank sum test between consecutive time points are represented by asterisks (* when <0.05). CSD: C-section delivery, VD: vaginal delivery. Fecal samples originating from VD infants are represented on the left side of each barplot and by green points, samples from CSD infants are represented on the right side of each barplot and by blue points.

**Fig. S6. Differences between delivery modes in relation to relative abundances of Bacteroidetes and *Bacteroides* spp..** (**A**) Relative abundance of the phylum *Bacteroidetes* and (**B**) the genus *Bacteroides* and according to delivery mode. The numbers of samples per collection time point are given at the top of the graph. Significant differences obtained by Wilcoxon rank sum test and according to delivery mode are represented by asterisks (* when <0.05; ** when <0.01). CSD: C-section delivery, VD: vaginal delivery. Fecal samples originating from VD infants are represented on the left side of each barplot and by green points, samples from CSD infants are represented on the right side of each barplot and by blue points.

**Fig. S7. Relative abundances of Bacteroidetes in children born at different gestational ages.** Relative abundance of the phylum *Bacteroidetes* according to gestational age at day 3 after birth. Significant differences obtained by the Kruskal-Wallis test and according to gestational age are represented by an asterisk (* when < 0.05). LP: late preterm (34-36 weeks), ET: early term (37-38 weeks), FT: full term (≥39 weeks), CSD: C-section delivery, VD: vaginal delivery. For additional information, fecal samples originating from VD infants are represented by green points, samples from CSD infants by blue points.

**List of Supplementary Tables**

**Table S1.** Milk feeding regime of the infants prior to the different sample collection time points.

**Table S2.** Results of Wilcoxon rank sum tests comparing the yields measured for the prokaryotic and fungal DNA at different collection time points.

**Table S3.** Results of Wilcoxon rank sum tests for prokaryotic diversity, evenness, richness and dissimilarity indices at different collection time points.

**Table S4.** Results of Wilcoxon rank sum tests for microeukaryotic diversity, evenness, richness and dissimilarity indices at different collection time points.

**Table S1. Milk feeding regime of the infants prior to the different sample collection time points.** Up to each collection time points, the infant was either purely breast milk fed (Breast), formula milk fed (Formula), received a combination of both breast and formula milk (Mixed) or was introduced to solid food (Weaning). If an infant has received formula or combined milk once, it was considered being fed combined milk for all subsequent time points. VD: vaginal delivery, CSD: C-section delivery.

| **VD** | | | | | | | | | |
| --- | --- | --- | --- | --- | --- | --- | --- | --- | --- |
| Collection time point/ Infant number | VD1 | VD2 | VD3 | VD4 | | VD5 | VD6 | VD7 | VD8 |
| Day 1 | Breast | Formula | Breast | Breast | | Breast | Breast | Breast | Breast |
| Day 3 | Breast | Combined | Combined | Breast | | Breast | Breast | Formula | Breast |
| Day 5 | Breast | Combined | Combined | Breast | | Breast | Breast | Formula | Breast |
| Day 28 | Breast | Combined | Combined | Breast | | Breast | Breast | Formula | Breast |
| Day 150 | Combined | Combined | Combined | Breast | | Formula | Breast | Formula | Breast |
| Day 365 | Weaning | Weaning | Weaning | Weaning | | Weaning | Weaning | Weaning | Weaning |
| **CSD** | | | | |  | | | | |
| Collection time point/ Infant number | CSD1 | CSD2 | CS3 | CS4 | | CS5 | CS6 | CS7 |  |
| Day 1 | Breast | Breast | Breast | Breast | | Breast | Breast | Breast |  |
| Day 3 | Breast | Breast | Breast | Breast | | Breast | Breast | Combined |  |
| Day 5 | Breast | Breast | Breast | Breast | | Breast | Breast | Combined |  |
| Day 28 | Breast | Breast | Breast | Breast | | Combined | Breast | Combined |  |
| Day 150 | Breast | Breast | Breast | Breast | | Combined | Breast | Combined |  |
| Day 365 | Weaning | Weaning | Weaning | Weaning | | Weaning | Weaning | Weaning |  |

**Table S2. Results of Wilcoxon rank sum tests comparing the yields measured for the prokaryotic and fungal DNA at different collection time points.** Only P-values below 0.05 are given.

| **16S** |  |  |  |  |  |  |
| --- | --- | --- | --- | --- | --- | --- |
| ng prokaryotic  DNA/mg of stool | Day 1 | Day 3 | Day 5 | Day 28 | Day 150 | Day 365 |
| Day 1 |  | 2.9E-03 | 1.4E-03 | 2.2E-04 | 4.4E-04 | 1.8E-04 |
| Day 3 |  |  |  |  |  | 7.0E-04 |
| Day 5 |  |  |  |  | 1.9E-02 | 1.9E-04 |
| Day 28 |  |  |  |  |  | 1.1E-04 |
| Day 150 |  |  |  |  |  | 3.8E-02 |
| Day 365 |  |  |  |  |  |  |
|  |  |  |  |  |  |  |
| **18S** |  |  |  |  |  |  |
| ng fungal DNA/mg of stool | Day 1 | Day 3 | Day 5 | Day 28 | Day 150 | Day 365 |
| Day 1 |  |  |  |  |  |  |
| Day 3 |  |  |  |  |  |  |
| Day 5 |  |  |  |  |  |  |
| Day 28 |  |  |  |  |  |  |
| Day 150 |  |  |  |  |  |  |
| Day 365 |  |  |  |  |  |  |

**Table S3. Results of Wilcoxon rank sum tests for prokaryotic diversity (Shannon’s diversity index), evenness (Pielou’s evenness index), richness (number of different OTUs) and dissimilarity (distance of the different collection time points to the respective latest collected time point according to Soerensen) indices at different collection time points.** Only P-values below 0.05 are given.

| Richness | Day 1 | Day 3 | Day 5 | Day 28 | Day 150 | Day 365 |
| --- | --- | --- | --- | --- | --- | --- |
| Day 1 |  | 1.6E-02 | 1.7E-03 | 9.2E-03 | 1.7E-02 | 1.3E-02 |
| Day 3 |  |  |  |  |  |  |
| Day 5 |  |  |  | 3.4E-02 |  |  |
| Day 28 |  |  |  |  |  |  |
| Day 150 |  |  |  |  |  |  |
| Day 365 |  |  |  |  |  |  |
|  |  |  |  |  |  |  |
| Shannon | Day 1 | Day 3 | Day 5 | Day 28 | Day 150 | Day 365 |
| Day 1 |  | 3.7E-02 | 1.7E-03 | 1.2E-02 |  |  |
| Day 3 |  |  |  |  | 1.6E-02 | 4.8E-03 |
| Day 5 |  |  |  | 1.5E-03 | 1.9E-04 | 8.7E-05 |
| Day 28 |  |  |  |  | 2.0E-02 | 1.8E-04 |
| Day 150 |  |  |  |  |  | 1.1E-02 |
| Day 365 |  |  |  |  |  |  |
|  |  |  |  |  |  |  |
| Pielou | Day 1 | Day 3 | Day 5 | Day 28 | Day 150 | Day 365 |
| Day 1 |  |  | 1.7E-03 | 1.7E-02 |  |  |
| Day 3 |  |  |  |  | 2.5E-02 | 4.8E-03 |
| Day 5 |  |  |  | 2.5E-03 | 2.5E-04 | 8.7E-05 |
| Day 28 |  |  |  |  | 1.4E-02 | 1.4E-04 |
| Day 150 |  |  |  |  |  | 5.8E-03 |
| Day 365 |  |  |  |  |  |  |
|  |  |  |  |  |  |  |
| Dissimilarity  index | Day 1 | Day 3 | Day 5 | Day 28 | Day 150 |  |
| Day 1 |  |  |  |  | 8.8E-03 |  |
| Day 3 |  |  |  |  |  |  |
| Day 5 |  |  |  |  |  |  |
| Day 28 |  |  |  |  |  |  |
| Day 150 |  |  |  |  |  |  |

**Table S4. Results of Wilcoxon rank sum tests for microeukaryotic diversity (Shannon’s diversity index), evenness (Pielou’s evenness index), richness (number of different OTUs) and dissimilarity (distance of the different collection time points to the respective latest collected time point according to Soerensen) indices at different collection time points.** Only P-values below 0.05 were considered significant.

| Richness | Day 1 | Day 3 | Day 5 | Day 28 | Day 150 | Day 365 |
| --- | --- | --- | --- | --- | --- | --- |
| Day 1 |  |  |  |  |  |  |
| Day 3 |  |  |  |  |  |  |
| Day 5 |  |  |  |  |  |  |
| Day 28 |  |  |  |  |  | 7.5E-03 |
| Day 150 |  |  |  |  |  |  |
| Day 365 |  |  |  |  |  |  |
|  |  |  |  |  |  |  |
| Shannon | Day 1 | Day 3 | Day 5 | Day 28 | Day 150 | Day 365 |
| Day 1 |  |  |  |  |  |  |
| Day 3 |  |  |  |  |  |  |
| Day 5 |  |  |  |  |  |  |
| Day 28 |  |  |  |  |  | 2.3E-02 |
| Day 150 |  |  |  |  |  |  |
| Day 365 |  |  |  |  |  |  |
|  |  |  |  |  |  |  |
| Pielou | Day 1 | Day 3 | Day 5 | Day 28 | Day 150 | Day 365 |
| Day 1 |  |  |  |  |  |  |
| Day 3 |  |  |  |  |  | 1.8E-02 |
| Day 5 |  |  |  |  |  |  |
| Day 28 |  |  |  |  |  |  |
| Day 150 |  |  |  |  |  |  |
| Day 365 |  |  |  |  |  |  |
|  |  |  |  |  |  |  |
| Dissimilarity  index | Day 1 | Day 3 | Day 5 | Day 28 | Day 150 |  |
| Day 1 |  |  |  |  |  |  |
| Day 3 |  |  |  |  |  |  |
| Day 5 |  |  |  |  |  |  |
| Day 28 |  |  |  |  |  |  |
| Day 150 |  |  |  |  |  |  |
